# Supplementary material for: Effectiveness of structured interventional strategy for middle-aged adolescence (SISMA-PA) for preventing atherosclerotic risk factors—A study protocol
Source: PLoS One. 2022 Jul 19;17(7):e0271599. doi: 10.1371/journal.pone.0271599 (PMC9295980; doi:10.1371/journal.pone.0271599)
Supplement: S2 File — (DOCX) [file pone.0271599.s002.docx]

**பெற்றோர் ஒப்புதல் படிவம்**

திட்ட தலைப்பு: "தேர்ந்தெடுக்கப்பட்ட பள்ளிகளில் நடுத்தர வயது இளம்பருவத்தில் இரத்த நாளங்களில் (தமனிகளில்) கொழுப்பு தகடு குவிப்பு அல்லது பெருந்தமனி தடிப்புத் தோல் அழற்சி ஆபத்து காரணிகளைத் தடுப்பதற்கான விழிப்புணர்வு நடவடிக்கையின் செயல்திறன்".

பங்கேற்புக்கான அழைப்பு:

அன்புள்ள பெற்றோர் / பாதுகாவலர், என் பெயர் திருமதி கோமதி முனுசாமி. நான் ஆந்திராவின் நெல்லூர், சிந்தாரெட்டி பாலம், நாராயணா செவிலியர் (நர்சிங்) கல்லூரி, சமூக சுகாதார செவிலியர் (நர்சிங்) துறையில் ஆராய்ச்சி அறிஞராகப் படிக்கிறேன். இளமை பருவத்தில் பெருந்தமனி தடிப்பு அபாயங்களைத் தடுப்பது பற்றிய விழிப்புணர்வு ஆராய்ச்சி ஆய்வு நடத்துகிறேன். இந்தப் படிவத்தின் நோக்கம் உங்கள் குழந்தைக்கு இந்த ஆராய்ச்சியில் பங்கேற்க ஒப்புதல் அளிப்பதா என்பதைத் தீர்மானிக்க உதவும் தகவலை உங்களுக்கு வழங்குவதாகும்.

இந்த ஆய்வு பற்றி முக்கிய தகவல்:

உலகெங்கிலும் இந்தியாவிலும் இரத்த நாளங்களில் (தமனிகளில்) கொழுப்பு தகடு குவிப்பு அல்லது பெருந்தமனி தடிப்புத் தோல் அழற்சியின் நிகழ்வு அதிகரித்து வருகிறது மற்றும் எதிர்காலத்தில் இருதய நோயை உருவாக்கும் வகையில் இப்போது இளைஞர்களிடையே வாழ்க்கை முறை மாற்றங்கள் உள்ளன. இந்த ஆய்வானது, பெருந்தமனி தடிப்பு ஆபத்து காரணிகள், உடல் செயல்பாடு, உணவுப் பழக்கம் மற்றும் உட்கார்ந்த செயல்பாடு போன்ற இளம் பருவத்தினரின் ஆரோக்கிய நடத்தைகள் பற்றிய அறிவைக் கண்டறியும்.

ஆய்வின் நோக்கம்:

இந்த ஆய்வின் நோக்கம் பெருந்தமனி தடிப்பு ஆபத்து காரணிகள் மற்றும் இளம் பருவத்தினரின் உடல்நலப் பயிற்சிகள், உணவுப் பழக்கம் மற்றும் உட்கார்ந்த செயல்பாடு போன்ற அறிவை கண்டறிவது ஆகும்.

ஆராய்ச்சியின் நடைமுறைகள்:

உங்கள் குழந்தை ஆராய்ச்சியில் பங்கேற்க நீங்கள் ஒப்புக்கொண்டால், உங்களது குழந்தைகளின் எடை, உயரம், உடல் நிறை குறியீடு, இடுப்பு சுற்றளவு, இரத்த அழுத்தம், இரத்த சர்க்கரை அளவு மற்றும் இரத்த கொழுப்பின் அளவு (இது இரண்டு முறை (முதல் மற்றும் 4 வது மாதத்தில்) போன்ற ஆரோக்கிய அளவீடுகள் எடுக்கப்படும். பெருந்தமனி தடிப்பு ஆபத்து காரணிகள் பற்றிய தகவல்கள் பவர் பாயிண்ட் விளக்கக்காட்சி மூலம் ஆராய்ச்சியாளரால் விளக்கப்படும். ஸ்கிப்பிங், வாக்கிங், ஜாகிங், ரன்னிங், டான்ஸ் மற்றும் சைக்கிள் பயிற்சி போன்ற உடல் செயல்பாடு உடற்கல்வி ஆசிரியரால் வழங்கப்படும். உடல் செயல்பாடுகளை மதிப்பிடுவதற்கு மாதத்திற்கு ஒரு வாரம் ஃபிட்னஸ் பேண்ட் ரிஸ்ட் வாட்ச் (பிடோமீட்டர்) வழங்கப்படும். உடல் செயல்பாடுகளின் நன்மைகள், ஆரோக்கியமான உணவுப் பழக்கங்கள் மற்றும் உட்கார்ந்த செயல்பாட்டைக் கட்டுப்படுத்துதல் போன்ற பெருந்தமனி தடிப்பு ஆபத்து காரணிகளைத் தடுக்க ஒரு கையேடு வழங்கப்படும்.

அபாயங்கள் மற்றும் பாதுகாப்புகள்:

முன்னறிவிக்கக்கூடிய அபாயங்கள் எதுவும் இல்லை. ஆய்வக தொழில்நுட்ப வல்லுநர்களால் இரத்த மாதிரிகள் சேகரிக்கப்படும். சுத்தமான ஊசி disposable syringes பயன்படுத்தப்படும், ஆல்கஹால் துடைப்பால் தோல் தளத்தை சுத்தம் செய்யப்படும். உங்கள் குழந்தையின் தகவல் மற்றவர்கள் அறியப்படாமல் இரகசியமாக இருக்கும்.

தன்னார்வ பங்கேற்பு:

இந்த ஆய்வில் உங்கள் குழந்தையின் பங்கேற்பு தன்னார்வமானது.

பங்கேற்பு காலம்:

உங்கள் குழந்தை 4 மாத காலத்திற்கு இந்த ஆராய்ச்சி ஆய்வில் பங்கேற்பார்.

ஆராய்ச்சி ஆய்வில் பங்கேற்பதன் நன்மைகள்:

உங்கள் குழந்தையின் உடல் எடையை குறைப்பது மற்றும் உங்கள் குழந்தையின் அளவீட்டு உயரம், எடை, உடல் நிறை குறியீடு, இடுப்பு சுற்றளவு, இரத்த அழுத்தம், இரத்த குளுக்கோஸ் மற்றும் இரத்தக் கொழுப்பு அளவுகள் ஆகியவற்றை அறிய முடியும். ஆய்வில் பங்கேற்பதன் மூலம் எதிர்காலத்தில் ஆபத்து காரணிகள் மற்றும் பெருந்தமனி தடிப்புத் தோல் அழற்சியைத் தடுப்பது பற்றியும் உங்கள் பிள்ளைக்குத் தெரியும்.

ஊக்கத்தொகை தகவல்:

இந்த ஆய்வின் முடிவில், பங்கேற்புக்காக உங்கள் பிள்ளை ஆராய்ச்சி முடிந்தவுடன் 25 ரூபாய் மதிப்புள்ள நோட்புக்கைப் பெறுவார். இரத்தக் கொழுப்பு மற்றும் சர்க்கரையை பரிசோதிப்பதற்கு பணம் செலுத்த வேண்டிய அவசியமில்லை மற்றும் ஆய்வக அறிக்கை (Lab report) உங்கள் குழந்தைக்கு வழங்கப்படும். உங்கள் குழந்தை பெருந்தமனி தடிப்புத் தோல் அழற்சியின் தடுப்பு அம்சங்களைப் பற்றிய அறிவைப் பெறுவர்.

தொடர்பு தகவல்:

ஆராய்ச்சி பற்றி ஏதேனும் சந்தேகங்கள் இருந்தால், என்னை 9789640804 என்ற எண்ணில் அழைக்கவும் அல்லது [**gomathilingeswaran@gmail.com**](mailto:gomathilingeswaran@gmail.com) இந்த மின்னஞ்சலை தொடர்பு கொள்ளவும்.

பெற்றோரின் ஆதாரம்:

கீழே கையொப்பமிடுவதன் மூலம், உங்கள் குழந்தை மேற்கண்ட ஆய்வில் பங்கேற்க ஒப்புதல் அளிக்கிறீர்கள்.

கையொப்பமிடுவதற்கு முன் உங்களுக்கு பொருந்தும் விருப்பத்தை சரிபார்த்து டிக் மார்க் (✓) வைக்கவும்:

☐ எனது குழந்தைக்கு ஆடியோ/வீடியோ டேப் செய்ய அனுமதி அளிக்கிறேன்.

☐ எனது குழந்தைக்கு ஆடியோ/வீடியோ எடுக்க நான் அனுமதி அளிக்கவில்லை.

☐ என் குழந்தைக்கு இரத்தக் கொழுப்பு மற்றும் குளுக்கோஸைச் சரிபார்க்க நான் அனுமதி அளிக்கிறேன்

☐ எனது குழந்தை இரத்தக் கொழுப்பு மற்றும் குளுக்கோஸைச் சரிபார்க்க நான் அனுமதி அளிக்கவில்லை

உங்கள் குழந்தையின் பெயர்: __________________________________

பெற்றோரின் / பாதுகாவலர் பெயர்: _______________________________________

பெற்றோர் / பாதுகாவலர் கையொப்பம்: ___________________________________

தேதி: ___________________________
