# Supplementary material for: Effectiveness of structured interventional strategy for middle-aged adolescence (SISMA-PA) for preventing atherosclerotic risk factors—A study protocol
Source: PLoS One. 2022 Jul 19;17(7):e0271599. doi: 10.1371/journal.pone.0271599 (PMC9295980; doi:10.1371/journal.pone.0271599)
Supplement: S3 File — (DOCX) [file pone.0271599.s003.DOCX]

**பங்கேற்பாளர் ஒப்புதல் படிவம்**

திட்ட தலைப்பு: "தேர்ந்தெடுக்கப்பட்ட பள்ளிகளில் நடுத்தர வயது இளம்பருவத்தில் இரத்த நாளங்களில் (தமனிகளில்) கொழுப்பு தகடு குவிப்பு அல்லது பெருந்தமனி தடிப்புத் தோல் அழற்சி ஆபத்து காரணிகளைத் தடுப்பதற்கான விழிப்புணர்வு நடவடிக்கையின் செயல்திறன்".

**நான் ஏன் இங்கே இருக்கிறேன்?**

என் பெயர் திருமதி கோமதி முனுசாமி. நான் ஆந்திராவின் நெல்லூர், சிந்தாரெட்டி பாலம், நாராயணா செவிலியர் (நர்சிங்) கல்லூரி, சமூக சுகாதார செவிலியர் (நர்சிங்) துறையில் ஆராய்ச்சி அறிஞராகப் படிக்கிறேன். இளமை பருவத்தில் பெருந்தமனி தடிப்பு அபாயங்களைத் தடுப்பது பற்றிய விழிப்புணர்வு ஆராய்ச்சி ஆய்வில் பங்கேற்க நான் உங்களை அழைக்கிறேன்.

இந்த ஆய்வு பற்றிய முக்கிய தகவல் என்ன?

நீங்கள் இந்த ஆய்வின் ஒரு பகுதியாக இருக்க விரும்புகிறீர்களா என்பதை முடிவு செய்ய இந்த ஆய்வின் சுருக்கம் பின்வருமாறு. இந்த ஆய்வின் நோக்கம் இரத்த நாளங்களில் (தமனிகளில்) கொழுப்பு தகடு குவிப்பு அல்லது பெருந்தமனி தடிப்புத் தோல் அழற்சி மற்றும் அதனை தடுப்பது பற்றிய விவரங்கள், உடல் பயிற்சி, நல்ல உணவுப் பழக்கம் மற்றும் உட்கார்ந்த செயல்பாட்டைக் கட்டுப்படுத்துதல் போன்ற ஆரோக்கிய நடைமுறைகளைக் கண்டறிந்து வழங்குவதாகும்.

இந்த ஆய்வு ஏன் செய்யப்படுகிறது?

இந்த ஆய்வின் நோக்கம் பெருந்தமனி தடிப்பு ஆபத்து காரணிகள் மற்றும் இளம் பருவத்தினரின் உடல்நலப் பயிற்சிகள், உணவுப் பழக்கம் மற்றும் உட்கார்ந்த செயல்பாடு போன்ற அறிவை கண்டறிவது ஆகும்.

நான் என்ன செய்ய வேண்டும்?

நீங்கள் ஆராய்ச்சியில் இருக்க முடிவு செய்தால்,

1. பெருந்தமனி தடிப்பு ஆபத்து காரணிகள் மற்றும் அவற்றின் தடுப்பு, உடல் செயல்பாடு, உணவு பழக்கம் மற்றும் உட்கார்ந்த நடத்தை பற்றிய கேள்விகளுக்கு பதிலளிக்க வேண்டும்.

2. உயரம், எடை, இடுப்பு சுற்றளவு, மற்றும் இரத்த அழுத்தம் ஆகியவற்றை அளவிட அனுமதிக்க வேண்டும். அளவீடு செய்யும் போது பாதுகாப்பு வழங்கப்படும்.

3. உங்களின் கொலஸ்ட்ரால் மற்றும் சர்க்கரை அளவை கண்டறிய ஒரு சிறிய அளவு இரத்தத்தை வழங்க வேண்டும். இது இரண்டு முறை (முதல் மற்றும் 4 வது மாதத்தில்) எடுக்கப்படும்.

4. பெருந்தமனி தடிப்பு ஆபத்து காரணிகள் மற்றும் அதன் தடுப்பு பற்றிய வகுப்பில் கலந்து கொள்ள வேண்டும். இது ஒரு மாதத்திற்கு ஒருமுறை 60 நிமிடங்கள் எடுக்கப்படும்.

5. மிதமான முதல் தீவிரமான உடற்பயிற்சி முறைகளைப் பின்பற்ற வேண்டும்: முதல் மாதம் வாரத்திற்கு ஒரு முறை 30 நிமிடங்கள் (உடல் வெப்பமடைதல் (warm up), ஸ்கிப்பிங், நடைபயிற்சி மற்றும் ஓய்வு காலம்), இரண்டாவது மாதம் வாரத்திற்கு இரண்டு முறை 45 நிமிடங்கள் (உடல் வெப்பமடைதல் (warm up), ஸ்கிப்பிங், நடைபயிற்சி, ஓட்டம், நடனம் மற்றும் ஓய்வு காலம்), மூன்றாவது மாதம் வாரத்திற்கு மூன்று முறை 60 நிமிடங்கள் (உடல் வெப்பமடைதல் (warm up), ஸ்கிப்பிங், நடைபயிற்சி, ஓடுதல், நடனம், சைக்கிள் ஓட்டுதல் மற்றும் ஓய்வு காலம்). உங்கள் உடல் செயல்பாடுகளை மதிப்பிடுவதற்கு மாதத்திற்கு ஒரு வாரம் ஃபிட்னஸ் பேண்ட் ரிஸ்ட் வாட்ச் (பிடோமீட்டர்) வழங்கப்படும்.

6. ஆரோக்கியமான உணவுப் பழக்க வழக்கம், தொலைக்காட்சி மற்றும் மொபைல் பார்ப்பதை குறைப்பது பற்றிய தகவல் கையேடு உங்களுக்கு வழங்கப்படும்.

7. உங்களின் உடல் செயல்பாடு, ஆரோக்கியமான உணவுப் பழக்கம் மற்றும் உட்கார்ந்த செயல்பாட்டைக் குறைப்பது பற்றிய தகவல்கள் பெற்றோருக்கு மாதம் ஒரு முறை செய்திமடலாக தரப்படும்.

8. நான் உங்களின் ஆடியோ அல்லது வீடியோ பதிவுக்குச் செல்கிறேன் என்றால், உங்கள் அனுமதியின்றி உங்களின் செயல்பாடுகளை நான் பதிவு செய்ய மாட்டேன்.

எனக்கு என்ன நன்மைகள்?

இந்த ஆய்வு உங்கள் உடல் எடையை அடித்தளத்திலிருந்து 5% - 10% குறைக்கச் செய்யும், மேலும் உங்கள் அளவீட்டு உயரம், எடை, உடல் நிறை குறியீட்டெண், இடுப்பு சுற்றளவு, இரத்த அழுத்தம், இரத்த குளுக்கோஸ் மற்றும் இரத்தம் கொழுப்பு அளவுகள் ஆகியவற்றை அறியலாம். ஆய்வில் பங்கேற்பதன் மூலம் எதிர்காலத்தில் இரத்த நாளங்களில் (தமனிகளில்) கொழுப்பு தகடு குவிப்பு அல்லது பெருந்தமனி தடிப்புத் தோல் அழற்சி நோய் ஆபத்து காரணிகளை தடுப்பது பற்றி நீங்கள் அறிந்து கொள்வீர்கள், அத்துடன் மற்றவர்களுக்கும் இது பற்றிய அறிவைப் பகிர்ந்துகொள்ள முடியும்.

நான் இந்த ஆய்வில் ஈடுபட முடிவு செய்தால் எனக்கு ஏதேனும் ஆபத்துகள் உள்ளதா?

முன்னறிவிக்கக்கூடிய அபாயங்கள் எதுவும் இல்லை. ஆய்வக தொழில்நுட்ப வல்லுநர்களால் இரத்த மாதிரிகள் சேகரிப்படும். சுத்தமான ஊசி disposable syringes பயன்படுத்தப்படும், ஆல்கஹால் துடைப்பால் தோல் தளத்தை சுத்தம் செய்யப்படும்.

எனது தகவல் எவ்வாறு பாதுகாக்கப்படும்?

உங்களின் தகவல் மற்றவர்கள் அறியப்படாமல் இரகசியமாக இருக்கும்; ஆய்வின் போது உங்களுக்கு ஐடி எண்கள் ஒதுக்கப்படும். உங்களின் பெயர், தொலைபேசி எண், முகவரி, பிறந்த தேதி மற்றும் மாணவர் ஐடி போன்ற தகவல்கள் இரகசியமாக இருக்கும். இந்த ஆய்வின் முடிவுகள், அறிக்கைகள், விளக்கக்காட்சிகள் அல்லது வெளியீடுகளில் CTRI (கிளினிக்கல் ட்ரையல் ரிஜிஸ்ட்ரி இந்தியா)க்கு தெரிவிக்கப்படும், ஆனால் உங்கள் பெயர் பயன்படுத்தப்படாது. ஆராய்ச்சியின் முடிவுகள் மொத்தமாக மட்டுமே பகிரப்படும். தகவல்கள் கணினியில் சேமிக்கப்படும், மேலும் தனிப்பட்ட விவரங்களை ஆய்வு செய்யும் ஆராய்ச்சியாளரால் மட்டுமே அணுக முடியும். ஆராய்ச்சி திட்டம் முடிந்த பிறகு 10 ஆண்டுகள் வரை தகவல் விவரங்கள் தக்கவைக்கப்படும். பிறகு டிஜிட்டல் கோப்புகளில் சேமிக்கப்பட்ட தகவல்கள் அழிக்கப்படும்.

நான் ஆராய்ச்சி ஆய்வில் இருக்க வேண்டுமா?

தேர்வு உங்களுடையது. இந்த ஆய்வில் உங்கள் பங்கேற்பு முற்றிலும் தன்னார்வமானது.

இந்த ஆய்வில் பங்கேற்பதற்கு ஈடாக எனக்கு ஏதாவது கிடைக்குமா?

இந்த ஆய்வின் முடிவில், பங்கேற்புக்காக உங்களுக்கு ஆராய்ச்சி முடிந்தவுடன் 100 ரூபாய் மதிப்புள்ள நோட்புக்கைப் பெறுவீர். இரத்தக் கொழுப்பு மற்றும் சர்க்கரையை பரிசோதிப்பதற்கு பணம் செலுத்த வேண்டிய அவசியமில்லை மற்றும் ஆய்வக அறிக்கை உங்களுக்கு வழங்கப்படும். உங்களுக்கு பெருந்தமனி தடிப்புத் தோல் அழற்சியின் தடுப்பு அம்சங்களைப் பற்றிய அறிவைப் பெறுவீர்.

எனக்கு கேள்விகள் இருந்தால் என்ன செய்வது?

ஆராய்ச்சி பற்றி ஏதேனும் சந்தேகங்கள் இருந்தால், என்னை 9789640804 என்ற எண்ணில் அழைக்கவும் அல்லது [**gomathilingeswaran@gmail.com**](mailto:gomathilingeswaran@gmail.com) இந்த மின்னஞ்சலை தொடர்பு கொள்ளவும்.

கீழே கையெழுத்திடுவதன் மூலம் நீங்கள் இந்தப் படிவத்தைப் படித்திருக்கிறீர்கள் என்றும் இந்த ஆராய்ச்சி ஆய்வில் நீங்கள் இருக்க விரும்புகிறீர்கள் என்றும் அர்த்தம்.

பங்கேற்பாளரின் பெயர் (உங்கள் பெயரை வரியில் எழுதுங்கள்): __________________________________

பங்கேற்பாளரின் கையொப்பம் (உங்கள் கையொப்பத்தை வரியில் வைக்கவும்): _____________________________

தேதி: ___________________________

படிவத்தை விளக்கிய நபரின் பெயர்: ______________________________________

இந்த படிவத்தை விளக்கிய நபரின் கையொப்பம்: ______________________________________

தேதி: ______________________________
