# Supplementary material for: Effectiveness of structured interventional strategy for middle-aged adolescence (SISMA-PA) for preventing atherosclerotic risk factors—A study protocol
Source: PLoS One. 2022 Jul 19;17(7):e0271599. doi: 10.1371/journal.pone.0271599 (PMC9295980; doi:10.1371/journal.pone.0271599)
Supplement: S4 File — (DOCX) [file pone.0271599.s004.docx]

**Questionnaire ID no:**

| **PART – I SURVEY INFORMATION** |
| --- |

| **Location and Date, Consent, ID** | | **Response** | **Code** |
| --- | --- | --- | --- |
| 1. | School Name |  | S1 |
| 2. | Type of school | Government-1/Private-2/Government Aid-3 | S2 |
| 3. | Interviewer ID |  | S3 |
| 4. | Date of Data collection | DD/MM/YYYY | S4 |
| 5. | Assent &Consent has been read and obtained | Yes 1  No 2 If No, End | S5 |
| 6. | Time of interview (24-hour clock) | **Hrs. Min** | S6 |
| 7. | Reg no and Grade of Student & Section |  | S7 |

| **PART – II DEMOGRAPHIC VARIABLES** |
| --- |

| **Questions** | | | **Response** | | | |  |
| --- | --- | --- | --- | --- | --- | --- | --- |
| 8. | Age of the student (in years) [circle appropriate] | | 14 15 16 17 | | | | D1 |
| 9. | Gender | | Male 1 Female 2 | | | | D2 |
| 10. | Which standard are you? [circle appropriate] | | 10 11 12 | | | | D3 |
| 11. | Type of family | | Nuclear -1/Joint – 2/Extended - 3 | | | | D4 |
| 12. | Religion | | Hindu-1/Christian- 2/Muslim-3 /Others- 4 | | | | D5 |
| 13. | Locality of Residence | | Rural-1/Semi-urban- 2/Urban- 3 | | | | D6 |
| 14. | Monthly income of family | | (in Rupees) | | | | D7 |
| 15. | Occupation of father | | Health Professional-1/Technical Professional-2/Skilled worker-3/Unskilled worker-4/Unemployed-5 | | | | D8 |
| 16. | Occupation of Mother | | Housewife-1/Health professional-2/  Teaching professional-3/Non-Professional-4/Laborer-5 | | | | D9 |
| 17. | Educational status of the father | | Profession or Honours-1/Graduate- 2/Intermediate or Diploma-3/High School-4/Middle School-5/Primary School-6/Illiterate-7 | | | | D10 |
| 18. | Educational status of the mother | | Profession or Honours-1/Graduate- 2/Intermediate or Diploma-3/High School-4/Middle School-5/Primary School-6/Illiterate-7 | | | | D11 |
| 19. | Family Medical History | | Diabetes-1/Hypertension-2/Obesity-3/Stroke-4/None-5 | | | | D12 |
| 20. | Order of Birth | | Firstborn-1/Second born-2/Third born & above-3 | | | | D13 |
| 21. | Source of information gained on the accumulation of fat inside an artery (Atherosclerosis) | | Mass media-1/Friends and relatives-2/Health workers/School programs-3/None-4 | | | | D14 |
| **PART – II OBSERVATION CHECKLIST TO ASSESS THE PREVALENCE OF ATHEROSCLEROSIS IN MIDDLE AGE ADOLESCENCE** | | | | | | | |
| 22. | | Height in (cm) | |  | | C1 | |
| 23. | | Weight in( kg) | |  | | C2 | |
| 24. | | BMI | |  | | C3 | |
| 25. | | Waist circumference in (cm) | |  | | C4 | |
| 26. | | Hip circumference in (cm) | |  | | C5 | |
| 27. | | Waist Hip Ratio | |  | | C6 | |
| 28. | | Blood pressure (mm of Hg) | |  | | C7 | |
| 29. | | Blood Glucose (mgs/dl) | |  | | C8 | |
| 30. | | **Lipid Profile Test** | | | | | |
| 30a. | | Serum Total Cholesterol (mgs/dl) | |  | C10 | | |
| 30b. | | Triglycerides | |  | C11 | | |
| 30c. | | HDL Cholesterol (Direct) | |  | C12 | | |
| 30d. | | Non HDL Cholesterol | |  | C13 | | |
| 30e. | | LDL Cholesterol | |  | C14 | | |
| 30f. | | VLDL Cholesterol | |  | C15 | | |
| 30g. | | Coronary Risk Ratio - I | |  | C16 | | |
| 30h | | Coronary Risk Ratio - II | |  | C17 | | |

| **PART – III QUESTIONNAIRE TO ASSESS THE KNOWLEDGE ON PREVENTION OF ATHEROSCLEROTIC RISK FACTORS AMONG MIDDLE AGE ADOLESCENCE** | | | | | | | | | | | | | | | | |
| --- | --- | --- | --- | --- | --- | --- | --- | --- | --- | --- | --- | --- | --- | --- | --- | --- |
| **General Information** | | | | | | | | | | | | | | | | |
| 31. | | | Accumulation of fat or cholesterol in the inner wall of the arteries is known as atherosclerosis | | | | | | | True-1/False-2 | | | K1 | | | |
| 32. | | | Accumulation of fat or cholesterol in the arteries is formed as a fatty plaque in an arteries | | | | | | | True-1/False-2 | | | K2 | | | |
| 33. | | | Blood vessel which carries oxygenated blood is known as the artery | | | | | | | True-1/False-2 | | | K3 | | | |
| 34. | | | Fatty plaque hardens arteries and makes arteries narrow, which prevents oxygenated blood flow to the heart and other body organs | | | | | | | True-1/False-2 | | | K4 | | | |
| 35. | | | Family history contributes to atherosclerosis (accumulation of fat/cholesterol in the inner wall of the arteries) | | | | | | | True-1/False-2 | | | K5 | | | |
| 36. | | | Atherosclerosis (accumulation of fat in the inner wall of the arteries) is most common among female | | | | | | | True-1/False-2 | | | K6 | | | |
| 37. | | | Atherosclerosis (accumulation of fat in the inner wall of the arteries) risk starts early by the age of 15 years | | | | | | | True-1/False-2 | | | K7 | | | |
| 38. | | | A healthy diet provides all the nutrients and energy in required amounts and proper proportions (e.g., whole grain cereals, pulses, legumes, green leaves, green and yellow vegetables, fruits). | | | | | | | True-1/False-2 | | | K8 | | | |
| 39. | | | Packaged or pre-prepared foods which are ready-to-eat and cook is known as junk food | | | | | | | True-1/False-2 | | | K9 | | | |
| 40. | | | Processed foods that are refined and used for cooking e.g., white rice, maida, husked dhals (without outer covering), white sugar, etc., are unhealthy diets. | | | | | | | True-1/False-2 | | | K10 | | | |
| 41. | | | Fast foods/ bakery foods (which are low in fiber and vitamins), foods high in fats (especially of animal origin), processed foods, junk foods are known as unhealthy diet | | | | | | | True-1/False-2 | | | K11 | | | |
| 42. | | | A diet rich in fat, salt, sugar, foods such as mutton, liver (animal origin), fried potato chips/samosa/pakoda, burger, pizza, ice cream, sugary and carbonated beverages, butter, ghee, etc., are healthy diet | | | | | | | True-1/False-2 | | | K12 | | | |
| 43. | | | Sitting for a long time in one place, watching television, using mobile and computers for fun/education/communication, chatting with friends, using a motor vehicle for travel (bus/bike/train) are known as a sedentary activity | | | | | | | True-1/False-2 | | | K13 | | | |
| 44. | | | Very little or lack of physical exercise is called physical inactivity | | | | | | | True-1/False-2 | | | K14 | | | |
| 45. | | | Breath awareness (BAW) and relaxation techniques are known as meditation | | | | | | | True-1/False-2 | | | K15 | | | |
| **Risk Factors** | | | | | | | | | | | | | | | | |
| 46. | | Eating junk, fast foods, and foods of animal origin lead to the accumulation of fat in the inner wall of the arteries | | | | | | | | True-1/False-2 | | | K16 | | | |
| 47. | | Junk foods, fast foods, and foods from animal origin, which contains low levels of fiber, vitamin, and minerals those foods are bad for the heart | | | | | | | | True-1/False-2 | | | K17 | | | |
| 48. | | Very little or lack of physical exercise decreases bodyweight that prevents accumulation of fat in the inner wall of the arteries | | | | | | | | True-1/False-2 | | | K18 | | | |
| 49. | | Overweight and obese increases the risk of atherosclerosis (accumulation of fat in the inner wall of the arteries) | | | | | | | | True-1/False-2 | | | K19 | | | |
| 50. | | Excessive coffee consumption increases blood pressure and elevates levels of bad cholesterol | | | | | | | | True-1/False-2 | | | K20 | | | |
| 51. | | The increased sugar level in the blood decreases the chance of developing atherosclerosis (accumulation of fat in the inner wall of the arteries) | | | | | | | | True-1/False-2 | | | K21 | | | |
| 52. | | High blood pressure increases the chance of developing atherosclerosis (accumulation of fat in the inner wall of the arteries) | | | | | | | | True-1/False-2 | | | K22 | | | |
| 53. | | The high level of good cholesterol in the blood decreases the risk of developing atherosclerosis (accumulation of fat in the inner wall of the arteries) | | | | | | | | True-1/False-2 | | | K23 | | | |
| 54. | | The high level of bad cholesterol in the blood is an important risk factor for the development of atherosclerosis (accumulation of fat in the inner wall of the arteries) | | | | | | | | True-1/False-2 | | | K24 | | | |
| 55. | | Infections are one of the risk factors for the development of atherosclerosis (accumulation of fat in the inner wall of the arteries) | | | | | | | | True-1/False-2 | | | K25 | | | |
| 56. | | Daytime sleeping is one of the risk factors for the development of atherosclerosis (accumulation of fat in the inner wall of the arteries) | | | | | | | | True-1/False-2 | | | K26 | | | |
| 57. | | Cigarette smoking and exposure to second-hand smoke (passive) decreases the chance for the development of atherosclerosis (accumulation of fat in the inner wall of the arteries) | | | | | | | | True-1/False-2 | | | K27 | | | |
| 58. | | Drinking large amounts of alcohol, which increases the risk of developing atherosclerosis (accumulation of fat in the inner wall of the arteries) | | | | | | | | True 1 False 2 | | | K28 | | | |
| **Symptoms** | | | | | | | | | | | | | | | | |
| 59. | | Difficulty breathing is a symptom of atherosclerosis (accumulation of fat in the inner wall of the arteries) | | | | | | | | True-1/False-2 | | | K29 | | | |
| 60. | | Chest pain is a symptom of atherosclerosis (accumulation of fat in the inner wall of the arteries) | | | | | | | | True-1/False-2 | | | K30 | | | |
| 61. | | Swelling of the hand and feet is a symptom of atherosclerosis (accumulation of fat in the inner wall of the arteries) | | | | | | | | True-1/False-2 | | | K31 | | | |
| 62. | | Paralysis of the face, arm, or leg is a symptom of atherosclerosis (accumulation of fat in the inner wall of the arteries) | | | | | | | | True-1/False-2 | | | K32 | | | |
| 63. | | Weakness and numbness of legs are the symptoms of atherosclerosis (accumulation of fat in the inner wall of the arteries) | | | | | | | | True-1/False-2 | | | K33 | | | |
| 64. | | Stroke is due to lack of blood supply to Brain | | | | | | | | True-1/False-2 | | | K34 | | | |
| 65. | | A heart attack occurs due to blood flow stopped to the heart muscle | | | | | | | | True-1/False-2 | | | K35 | | | |
| 66. | | Impotence (difficulty in maintaining an erection of the penis) is a symptom of atherosclerosis (accumulation of fat in the inner wall of the arteries) | | | | | | | | True-1/False-2 | | | K36 | | | |
| **Diagnosis** | | | | | | | | | | | | | | | | |
| 67. | | Checking blood cholesterol is an investigation for atherosclerosis (accumulation of fat in the inner wall of the arteries) | | | | | | | | True-1/False-2 | | | K37 | | | |
| 68. | | Angiography is a special X-ray to show the inside of arteries in Heart Disease to detect early diagnosis of atherosclerosis (accumulation of fat in the inner wall of the arteries) | | | | | | | | True-1/False-2 | | | K38 | | | |
| **Prevention** | | | | | | | | | | | | | | | | |
| 69. | | Green leaves such as mustard leaves, drumstick leaves, fenugreek leaves, coriander leaves, etc., helps to prevent the development of atherosclerosis (accumulation of fat in the inner wall of the arteries) | | | | | | | | True-1/False-2 | | | K39 | | | |
| 70. | | Green vegetables such as beans, cabbage, bitter gourd, bottle gourd, ridge gourd, snake gourd, cucumber, etc., helps to prevent the development of atherosclerosis (accumulation of fat in the inner wall of the arteries) | | | | | | | | True-1/False-2 | | | K40 | | | |
| 71. | | Steamed and boiled vegetables are very healthier than fried vegetables | | | | | | | | True-1/False-2 | | | K41 | | | |
| 72. | | Intake of Fiber or roughage rich diet 30 gm per day, which helps in slowing down the absorption of sugar and fats into the blood and prevent the development of atherosclerosis (accumulation of fat in the inner wall of the arteries) | | | | | | | | True-1/False-2 | | | K42 | | | |
| 73. | | Increasing intake of fruits such as guava, gooseberry (amla), pomegranate, oranges, lemon, pineapple, avocado, etc., will reduce the risk of development of atherosclerosis (accumulation of fat in the inner wall of the arteries) | | | | | | | | True-1/False-2 | | | K43 | | | |
| 74. | | Reduce intake of oily, salty, sour, and spicy food items to reduce the risk of development of atherosclerosis (accumulation of fat in the inner wall of the arteries) | | | | | | | | True-1/False-2 | | | K44 | | | |
| 75. | | Intake of a low salt diet (1 teaspoon or 16 pinches or less than 5 grams per day) can prevent the development of atherosclerosis (accumulation of fat in the inner wall of the arteries) | | | | | | | | True-1/False-2 | | | K45 | | | |
| 76. | | Processed, fast and Junk food can prevent the development of atherosclerosis (accumulation of fat in the inner wall of the arteries) | | | | | | | | True-1/False-2 | | | K46 | | | |
| 77. | | Increasing consumption of refined cereals, high fat/oily foods, and sugary foods increase the risks of developing atherosclerosis. | | | | | | | | True-1/False-2 | | | K47 | | | |
| 78. | | Increase intake of fresh fruits, vegetables, whole grains, and whole pulses, millets, legumes decreases the chance of accumulation of fat in the inner wall of the arteries | | | | | | | | True-1/False-2 | | | K48 | | | |
| 79. | | Eating a balanced, healthy diet increases the chance of accumulation of fat in the inner wall of the arteries | | | | | | | | True-1/False-2 | | | K49 | | | |
| 80. | | For maintaining a healthy heart, daily food intake should include one quarter of fruits; one-quarter of vegetables; one-quarter of carbohydrates; One-quarter consisting of milk and dairy, meat, fish, and alternatives such as millets and pulses; the smallest portion of fats and sugary food. | | | | | | | | True-1/False-2 | | | K50 | | | |
| 81. | | Individuals when they are stressed, tend to have irregular eating habits and to eat more junk food (samosas, chips, pups) | | | | | | | | True-1/False-2 | | | K51 | | | |
| 82. | | The use of lukewarm water for drinking increases the chances of accumulation of fat in the inner wall of the arteries | | | | | | | | True-1/False-2 | | | K52 | | | |
| 83. | | Doing regular physical activity of 30 minutes for at least 5 days a week prevents the development of atherosclerosis (accumulation of fat in the inner wall of the arteries) | | | | | | | | True-1/False-2 | | | K53 | | | |
| 84. | | Daily 30 minutes of brisk morning walking, which prevents excessive weight gain and the chance of accumulation of fat in the inner wall of the arteries | | | | | | | | True-1/False-2 | | | K54 | | | |
| 85. | | A person who is overweight /obese needs to reduce the weight until they reach ideal/normal weight and it will reduce the risk of accumulation of fat in the inner wall of the arteries | | | | | | | | True-1/False-2 | | | K55 | | | |
| 86. | | Maintaining a healthy weight increases the risk of fat accumulation in the inner wall of the arteries | | | | | | | | True-1/False-2 | | | K56 | | | |
| 87. | | Doing regular physical exercise decreases the risk of stroke and improves control of blood glucose | | | | | | | | True-1/False-2 | | | K57 | | | |
| 88. | | Doing regular physical exercise, which reduces blood pressure and improves good cholesterol | | | | | | | | True-1/False-2 | | | K58 | | | |
| 89. | | Exercise is a healthy way to reduce and manage stress (e.g., walking, jogging, running, skipping, dancing) | | | | | | | | True-1/False-2 | | | K59 | | | |
| 90. | | Average 6 – 8 hours of sleep per day for an individual can prevent the risk of development of atherosclerosis (accumulation of fat in the inner wall of the arteries) | | | | | | | | True-1/False-2 | | | K60 | | | |
| 91. | | Sedentary activity affects health badly and increases the risk of atherosclerosis (accumulation of fat in the inner wall of the arteries) | | | | | | | | True-1/False-2 | | | K61 | | | |
| 92. | | Watching TV while eating is a good habit to maintain a healthy heart | | | | | | | | True-1/False-2 | | | K62 | | | |
| 93. | | Doing meditation and Yoga prevents the development of atherosclerosis (accumulation of fat in the inner wall of the arteries) | | | | | | | | True-1/False-2 | | | K63 | | | |
| 94. | | Doing yoga is beneficial for physical fitness, musculoskeletal function, and heart health | | | | | | | | True-1/False-2 | | | K64 | | | |
| 95. | | A regular medical check-up is needed for parents who have a history of heart disease to prevent atherosclerosis (accumulation of fat in the inner wall of the arteries) | | | | | | | | True-1/False-2 | | | K65 | | | |
| **PART – IV HEALTH BEHAVIOR PRACTICE ON PREVENTION AMONG MIDDLE AGE ADOLESCENCE** | | | | | | | | | | | | | | | | |
| **Section A: Physical Activity Questionnaire among Middle Age Adolescence** | | | | | | | | | | | | | | | | |
| 96. | Physical activity in your spare time: Have you done any of the following activities in the past 7 days (last week)? If yes, how many times?  No 1-2 3-4 5-6 7 times or more  Skipping ........................................... 􀂁 􀂁 􀂁 􀂁 􀂁  Walking for exercise ......................... 􀂁 􀂁 􀂁 􀂁 􀂁  Running ......................................... 􀂁 􀂁 􀂁 􀂁 􀂁  Dance……….................................. 􀂁 􀂁 􀂁 􀂁 􀂁  Bicycling .........................................􀂁 􀂁 􀂁 􀂁 􀂁 | | | | | | | | | | | | | | E1 | |
| 97. | In the last 7 days, during your physical education (PE) classes, how often were you very active (playing hard, running, jumping, throwing)? (Check one only.)  I don’t do PE .....................................................…... 􀂁  Hardly ever .............................................................. 􀂁  Sometimes ............................................................... 􀂁  Quite often ............................................................... 􀂁  Always ..................................................................... 􀂁 | | | | | | | | | | | | | | E2 | |
| 98. | In the last 7 days, what did you normally do *at lunch* (besides eating lunch)? (Check one only.)  Sat down (talking, reading, doing schoolwork).…....... 􀂁   Stood around or walked around ................................. 􀂁   Ran or played a little bit .............................................. 􀂁   Ran around and played quite a bit .............................. 􀂁   Ran and played hard most of the time ........................ 􀂁  | | | | | | | | | | | | | | E3 | |
| 99. | In the last 7 days, on how many days *right after school*, did you do sports, dance, or play games in which you were very active? (Check one only.)  None .................................................................……􀂁  1 time last week ....................................................... 􀂁  2 or 3 times last week .............................................. 􀂁  4 times last week ..................................................... 􀂁  5 times last week..................................................... 􀂁 | | | | | | | | | | | | | | E4 | |
| 100. | In the last 7 days, on how many *evenings* did you do sports, dance, or play games in which you were very active? (Check one only.)  None ........................................................................ 􀂁  1 time last week ....................................................... 􀂁  2 or 3 times last week .............................................. 􀂁  4 or 5 last week ........................................................ 􀂁  6 or 7 times last week .............................................. 􀂁 | | | | | | | | | | | | | | E5 | |
| 101. | *On the last weekend*, how many times did you do sports, dance, or play games in which you were very active? (Check one only.)  None ........................................................................ 􀂁  1 time ....................................................................... 􀂁  2 — 3 times ............................................................. 􀂁  4 — 5 times ............................................................. 􀂁  6 or more times ........................................................ 􀂁 | | | | | | | | | | | | | | E6 | |
| 102. | Which *one* of the following describes you best for the last 7 days? Read *all five* statements before deciding on the *one* answer that describes you.  A. All or most of my free time was spent doing things that involve little physical effort .................................................................................................………………………..􀂁  B. I sometimes (1 — 2 times last week) did physical things in my free time  (e.g. played sports, went running, swimming, bike riding, did aerobics) .......…………􀂁  C. I often (3 — 4 times last week) did physical things in my free time ..........……….. 􀂁  D. I quite often (5 — 6 times last week) did physical things in my free time ………...􀂁  E. I very often (7 or more times last week) did physical things in my free time …….. 􀂁 | | | | | | | | | | | | | | E7 | |
| 103. | Mark how often you did the physical activity (like playing sports, games, doing dance, or any other physical activity) for each day last week.  None Little bit Medium Often Very often  Monday ....................... 􀂁  􀂁 􀂁   Tuesday ...................... 􀂁  􀂁 􀂁   Wednesday ................. 􀂁  􀂁 􀂁   Thursday ..................... 􀂁  􀂁 􀂁   Friday .......................... 􀂁  􀂁 􀂁   Saturday ...................... 􀂁  􀂁 􀂁   Sunday ........................ 􀂁  􀂁 􀂁  | | | | | | | | | | | | | | E8 | |
| **Section B: Food Habit Questionnaire among Middle Age Adolescence** | | | | | | | | | | | | | | | | |
| 104. | I usually avoid eating fried foods | | | | | | Yes 1 No 2 | | | | | | | F1 | | |
| 105. | I usually skip breakfast to maintain my weight | | | | | | Yes 1 No 2 | | | | | | | F2 | | |
| 106. | I make sure I eat at least one serving of vegetables or vegetable salad a day | | | | | | Yes 1 No 2 | | | | | | | F3 | | |
| 107. | I make sure I eat at least one serving of fruit a day | | | | | | Yes 1 No 2 | | | | | | | F4 | | |
| 108. | I try to keep my overall fat intake down | | | | | | Yes 1 No 2 | | | | | | | F5 | | |
| 109. | I try to keep my overall sugar intake down | | | | | | Yes 1 No 2 | | | | | | | F6 | | |
| 110. | I often buy chocolates or cakes to eat | | | | | | Yes 1 No 2 | | | | | | | F7 | | |
| 111. | I avoid eating lots of samosas, pups, paani puri’s, burgers, and pizza | | | | | | Yes 1 No 2 | | | | | | | F8 | | |
| 112. | It’s a good habit to eat plenty of fruits and vegetables | | | | | | Yes 1 No 2 | | | | | | | F9 | | |
| 113. | I usually eat at least one serving of vegetables (excluding potatoes) or vegetable salad with my evening meals | | | | | | Yes 1 No 2 | | | | | | | F10 | | |
| 114. | When I am buying a soft drink, I usually choose a less sugary diet drink | | | | | | Yes 1 No 2 | | | | | | | F11 | | |
| 115. | If I have a packed lunch, I usually include some chips and/or chocolate | | | | | | Yes 1 No 2  I never have a packed lunch 3 | | | | | | | F12 | | |
| 116. | If I am buying chips/crisps, I often choose a low-fat brand | | | | | | Yes 1 No 2  I never buy chips/crisps 3 | | | | | | | F13 | | |
| 117. | If I am having lunch away from home, I often choose a low-fat diet | | | | | | Yes 1 No 2  I never have lunch away from home 3 | | | | | | | F14 | | |
| 118. | I rarely buy and eat food from outside | | | | | | Yes 1 No 2 | | | | | | | F15 | | |
| 119. | I often eat sweet snacks between meals | | | | | | Yes 1 No 2 | | | | | | | F16 | | |
| 120. | When I have a snack between meals, I often choose fruit | | | | | | Yes 1 No 2  I never eat snacks between meals 3 | | | | | | | F17 | | |
| 121. | When I put butter or ghee or jam on bread, I usually spread it thinly | | | | | | Yes 1 No 2  I never have butter or ghee or jam on bread 3 | | | | | | | F18 | | |
| 122. | I eat at least 3 servings of fruit most days | | | | | | Yes 1 No 2 | | | | | | | F19 | | |
| 123. | I generally try to have a healthy diet | | | | | | Yes 1 No 2 | | | | | | | F20 | | |
| 124 | If I am having dinner at home, I try to have food something low in fat | | | | | | Yes 1 No 2  I don’t eat dessert 3 | | | | | | | F21 | | |
| 125. | If I have dinner at a restaurant/hotel, I usually choose healthy food | | | | | | Yes 1 No 2  I never have dinner in a restaurant or hotel 3 | | | | | | | F22 | | |
| 126. | After eating dinner I usually go to bed after one hour | | | | | | Yes 1 No 2 | | | | | | | F23 | | |
| **Section C: Sedentary Activity Questionnaire among Middle Age Adolescence** | | | | | | | | | | | | | | | | |
| 127. | | | | Write down how long you spend doing the following activities before and after school each day (Weekdays). | **Monday** | | | **Tuesday** | **Wednes-**  **Day** | | **Thursday** | **Friday** | | | |  |
|  |  |  |  |  | Hrs-Mts | | | Hrs-Mts | Hrs-Mts | | Hrs-Mts | Hrs-Mts | | | |  |
|  |  |  |  | 1. Watching TV/video |  | | |  |  | |  |  | | | | S1 |
|  |  |  |  | 1. Mobile use for games |  | | |  |  | |  |  | | | | S2 |
|  |  |  |  | 1. Mobile use for education |  | | |  |  | |  |  | | | | S3 |
|  |  |  |  | 1. Computer use for fun |  | | |  |  | |  |  | | | | S4 |
|  |  |  |  | 1. Computer use for homework |  | | |  |  | |  |  | | | | S5 |
|  |  |  |  | 1. Reading for fun |  | | |  |  | |  |  | | | | S6 |
|  |  |  |  | 1. Active commuting to school (walking/cycling/bike/bus) |  | | |  |  | |  |  | | | | S7 |
|  | | | | 1. Sitting around (chatting with friends/ speaking on the phone/chilling |  | | |  |  | |  |  | | | | S8 |
| 128. | | | | Write down how long you spend doing the following activities on the weekend. | | **Saturday** | | | | | **Sunday** | | | | |  |
|  |  |  |  |  |  | Hrs-Mts | | | | | Hrs-Mts | | | | |  |
|  |  |  |  | 1. Watching TV/video | |  | | | | |  | | | | | S9 |
|  |  |  |  | 1. Mobile use for games | |  | | | | |  | | | | | S10 |
|  |  |  |  | 1. Mobile use for education | |  | | | | |  | | | | | S11 |
|  |  |  |  | 1. Computer use for fun | |  | | | | |  | | | | | S12 |
|  |  |  |  | 1. Computer use for homework | |  | | | | |  | | | | | S13 |
|  |  |  |  | 1. Reading for fun | |  | | | | |  | | | | | S14 |
|  |  |  |  | 1. Active commuting to school (walking/cycling/bike/bus) | |  | | | | |  | | | | | S15 |
|  | | | | 1. Sitting around (chatting with friends/speaking on the phone/chilling | |  | | | | |  | | | | | S16 |
