# Supplementary material for: Effectiveness of structured interventional strategy for middle-aged adolescence (SISMA-PA) for preventing atherosclerotic risk factors—A study protocol
Source: PLoS One. 2022 Jul 19;17(7):e0271599. doi: 10.1371/journal.pone.0271599 (PMC9295980; doi:10.1371/journal.pone.0271599)
Supplement: S5 File — (PDF) [file pone.0271599.s005.pdf]

Clinical Trial Details (PDF Generation Date :- Mon, 31 Jan 2022 15:03:56 GMT)

**CTRI Number**	CTRI/2021/03/032271 [Registered on: 24/03/2021] - **Trial Registered Prospectively**
**Last Modified On**	31/01/2022
**Post Graduate Thesis**	Yes
**Type of Trial**	Interventional
**Type of Study**	Preventive<br>Screening
**Study Design**	Other
**Public Title of Study**	Organized Interventional approach in teaching, physical exercise, healthy dietary pattern for middle age school going adolescence (15-17 years) to reduce the risk of accumulation of fat in arteries (Atherosclerosis)
**Scientific Title of Study**	Effectiveness of Structured Interventional Strategy on Prevention of Atherosclerotic risk factors among Middle Age Adolescence in selected Schools

Secondary IDs if Any

Secondary ID	Identifier
NIL	NIL

Details of Principal Investigator or overall Trial Coordinator (multi-center study)

Details of Principal Investigator	
**Name**	Gomathi Munusamy
**Designation**	Professor
**Affiliation**	Narayana College of Nursing
**Address**	Department of Community Health Nursing, Teaching Faculty in Community Health Nursing, Room No. 4, Narayana College of Nursing, Chinthareddypalem, Nellore, Andhra Pradesh Department of Community Health Nursing, Teaching Faculty in Community Health Nursing, Room No. 4, Narayana College of Nursing, Chinthareddypalem, Nellore, Andhra Pradesh<br>Nellore<br>ANDHRA PRADESH<br>524003<br>India
**Phone**	9618478803
**Fax**	
**Email**	gomathilingeswaran2678@gmail.com

Details Contact Person (Scientific Query)

Details Contact Person (Scientific Query)	
**Name**	Dr Ramesh Shanmugam
**Designation**	Professor
**Affiliation**	Sree Narayana College of Nursing
**Address**	Department of Medical Surgical Nursing, Teaching Faculty in Medical Surgical Nursing, Room No. 1, Sree Narayana College of Nursing, Chinthareddypalem, Nellore, Andhra Pradesh Department of Medical Surgical Nursing, Teaching Faculty in Medical Surgical Nursing, Room No. 1, Sree Narayana College of Nursing, Chinthareddypalem, Nellore, Andhra Pradesh<br>Nellore<br>ANDHRA PRADESH<br>524003<br>India
**Phone**	09789640804
**Fax**	
**Email**	rshanmugam704@gmail.com

Details Contact Person (Public Query)

Details Contact Person (Public Query)	
**Name**	Gomathi Munusamy

**Designation**	Professor
**Affiliation**	Narayana College of Nursing
**Address**	Department of Community Health Nursing, Teaching Faculty in Community Health Nursing, Room No. 4, Narayana College of Nursing, Chinthareddypalem, Nellore, Andhra Pradesh Department of Community Health Nursing, Teaching Faculty in Community Health Nursing, Room No. 4, Narayana College of Nursing, Chinthareddypalem, Nellore, Andhra Pradesh<br>Nellore<br>ANDHRA PRADESH<br>524003<br>India
**Phone**	9618478803
**Fax**	
**Email**	gomathilingeswaran2678@gmail.com

Source of Monetary or Material Support

Source of Monetary or Material Support
> Gomathi Munusamy Self Funding, Professor, Department of Community Health Nursing, Narayana College of Nursing, Chintareddypalem, Nellore, Andhra Pradesh,Pincode-524003

Primary Sponsor

Primary Sponsor Details	
**Name**	Gomathi Munusamy
**Address**	Narayana College of Nursing,Chintareddy Palem,Nellore,Andhra Pradesh
**Type of Sponsor**	Other [Self- sponsor]

Details of Secondary Sponsor

Name	Address
Gomathi Munusamy Self Funding	Narayana College of Nursing,Chintareddy Palem,Nellore,Andhra Pradesh

Countries of Recruitment

List of Countries
India

Sites of Study

Name of Principal Investigator	Name of Site	Site Address	Phone/Fax/Email
Mrs Kavitha M	Arakkonam	District Educational Offi cer,Arakkonam,Vellore District-631003<br>Vellore<br>TAMIL NADU	9442075481<br><br>i.kavitha@yahoo.com
Mr Y Simon Ponnaih	CSI Central Higher Secondary School	Education Department,SH 58,Arakkonam, Arokkonam Taluk,Vellore Division and District(Ranipet District),pincode-631001, Room No: 03<br>Vellore<br>TAMIL NADU	07598870656<br><br>csicentralhssarakkonam@gmail.com
Mr B Peter Gnanasekaran	CSI ST Andrews Higher Secondary School	Education Department, Andrews Street, Arakkonam, Arokkonam Taluk,Vellore Division and District(Ranipet District),Pincode - 631001 Room No: 03<br>Vellore<br>TAMIL NADU	09444888357<br><br>andrewshm@rediffmail.com

Mrs A Buelah Ambika	Government Higher Secondary School	Education Department, School Street, Mosur Village, Mosur, Arokkonam Taluk,Vellore Division and District(Ranipet District), Pincode-631004, Room No: 02 Vellore TAMIL NADU	09444162608<br><br>ghssmosurakm@gmail.com

Details of Ethics Committee

Name of Committee	Approval Status	Date of Approval	Is Independent Ethics Committee?
C.S.I. ST. Andrews Hr.Sec. School, Arakkonam	Approved	27/01/2021	No
CSI Central Hr. Sec School, Arakkonam	Approved	27/01/2021	No
District Educational Officer, Arakkonam	Approved	12/12/2019	No
Govt. Hr. Sec. School, Mosur	Approved	01/02/2021	No
Narayana College of Nursing	Approved	26/08/2019	No

Regulatory Clearance Status from DCGI

Status	Date
Not Applicable	No Date Specified

Health Condition / Problems Studied

Health Type	Condition
Healthy Human Volunteers	prevention and screening related to atherosclerotic risk factors

Intervention / Comparator Agent

Type	Name	Details
Intervention	Structured interventional strategy, Physical activity, reduction in sedentary activity and healthy dietary habit tips.	Research approach: Quantitative Research Approach Research design: Pre-experimental one group pre-post test time series research design. O1 x1 O2 x2 O3 x3 O4 O1 - Observation at the pre-test (Baseline) x1 - Structured interventional strategy, Physical activity (skipping and walking including warm-up and rest) for 30 minutes, reduction in sedentary activity and healthy dietary habit tips by booklet and newsletter to parents. O2 - Observation after 4 weeks x2 - Structured interventional strategy, Physical activity (skipping, walking, running, and dancing including warm-up and rest) for 45 minutes, reduction in sedentary activity and healthy dietary habit tips by booklet and newsletter to parents. O3 - Observation after 8 weeks x3 - Structured interventional strategy, Physical activity (skipping, walking,

			running, dancing, and bicycling including warm-up and rest) for 60 minutes, reduction in sedentary activity and healthy dietary habit tips by booklet and newsletter to parents. O4 - Observation after 12 weeks Interventional components Dose Description Students Structured interventional strategy Monthly once for 3 months x 60 minutes Participants will attend video and power point presentation on prevention of Atherosclerotic risk factors delivered by the researcher. Enhanced physical activity session 1st Phase (1 – 4 weeks): Skipping and walking including warm-up and resting for 30 minutes once in a week. 2nd Phase (5 – 8 weeks): Skipping, walking, running, and dancing including warm-up and resting for 45 minutes twice a week 3rd Phase (9 – 12 weeks): skipping, walking, running, dancing, and bicycling including warm-up and resting for 60 minutes thrice a week Sport sessions will be delivered by physical education teachers at the study schools. Behavioral messages will be reinforced during the cool-down period. Sedentary activity Monthly once for 3 months x 30 minutes Lesson with Information booklet: ? Encourage for active transport to/from school; ? Information about what and how to change/reduce screen time, ? Be active with friends and family. Dietary Pattern Monthly once for 3 months x 30 minutes Lesson with Information booklet: ? Nutrients to build a healthy body; ? Healthy food choices to prevent atherosclerosis risk factors; ? Increase fruits and vegetables intake; ? Cut, serve, taste and eat fruits and vegetables with class mates and family members, ? Drink more water, decrease sugar sweetened beverages, and junk foods. Parents Newsletter Monthly once for 3 months Parents of study participants will receive three newsletters containing information on the potential consequences of

		excessive screen-use among Middle age adolescents, strategies for reducing screen-based recreation in the home, and tips for healthy diet. They will also be provided with their adolescents baseline information.
Comparator Agent	NIL	NIL
Intervention	Structured interventional strategy, Physical activity, reduction in sedentary activity and healthy dietary habit tips.	O2 - Observation after 4 weeks x2 - Structured interventional strategy, Physical activity (skipping, walking, running, and dancing including warm-up and rest) for 45 minutes, reduction in sedentary activity and healthy dietary habit tips by booklet and newsletter to parents. O3 - Observation after 8 weeks x3 - Structured interventional strategy, Physical activity (skipping, walking, running, dancing, and bicycling including warm-up and rest) for 60 minutes, reduction in sedentary activity and healthy dietary habit tips by booklet and newsletter to parents. O4 - Observation after 12 weeks Interventional components Dose Description Students Structured interventional strategy Monthly once for 3 months x 60 minutes Participants will attend video and power point presentation on prevention of Atherosclerotic risk factors delivered by the researcher. Enhanced physical activity session 1st Phase (1 – 4 weeks): Skipping and walking including warm-up and resting for 30 minutes once in a week. 2nd Phase (5 – 8 weeks): Skipping, walking, running, and dancing including warm-up and resting for 45 minutes twice a week 3rd Phase (9 – 12 weeks): skipping, walking, running, dancing, and bicycling including warm-up and resting for 60 minutes thrice a week Sport sessions will be delivered by physical education teachers at the study schools. Behavioral messages will be reinforced during the cool-down period. Sedentary activity Monthly once for 3 months x 30 minutes

Inclusion Criteria

Inclusion Criteria	
**Age From**	15.00 Year(s)

**Age To**	17.00 Year(s)
**Gender**	Both
**Details**	1 Middle age adolescence who are studying in higher secondary schools aged between 15-17 years at Arakonam. 2 Middle age adolescence who are willing to participate in the study 3 Able to understand English and Tamil 4 Both genders are included in the study 5 BMI above 50th percentile

Exclusion Criteria

**Exclusion Criteria**	
**Details**	1 Middle age adolescence who are sick<br>2 Middle age adolescence who are practicing in weight management<br>3 Middle age adolescence whose sibling or relatives studying in other schools will be selected for the study<br>4 Middle age adolescence who is below 50th percentile or underweight

**Method of Generating Random Sequence**	Stratified randomization
**Method of Concealment**	Not Applicable
**Blinding/Masking**	Not Applicable

Primary Outcome

Outcome	Timepoints
BMI and physical activity	It will be assessed at 4th week, 8th week, and 12th week post intervention are BMI and physical activity

Secondary Outcome

Outcome	Timepoints
Dietary intake and sedentary activity will be assessed at 4th week, 8th week, and 12th week post intervention.	Dietary intake and sedentary activity will be assessed at 4th week, 8th week, and 12th week post intervention.
Dietary intake and sedentary activity	4th week, 8th week, and 12th week post intervention.

**Target Sample Size**	**Total Sample Size=**150<br>**Sample Size from India=**150<br>**Final Enrollment numbers achieved (Total)=**Applicable only for Completed/Terminated trials<br>**Final Enrollment numbers achieved (India)=**Applicable only for Completed/Terminated trials
**Phase of Trial**	N/A
**Date of First Enrollment (India)**	15/07/2021
**Date of First Enrollment (Global)**	No Date Specified
**Estimated Duration of Trial**	**Years=**0<br>**Months=**6<br>**Days=**0
**Recruitment Status of Trial (Global)**	Not Applicable
**Recruitment Status of Trial (India)**	Not Yet Recruiting
**Publication Details**	NIL

Brief Summary

Title: Effectiveness of Structured Interventional Strategy on Prevention of Atherosclerotic risk factors among Middle Age Adolescence in selected Schools

Background: Atherosclerotic risk factors are starting to increase in early childhood and adolescent which predisposes individuals to an increased risk of morbidity and mortality directly and indirectly associated with co-morbidity and poor lifestyle. Schools are the central focuses of interventions aiming the preventive strategy have not yet provided clear evidence of strategies to reduce prevalence of Atherosclerosis.

Purpose: The purpose of this study paper is to report the rationale and study protocol to evaluate the effect of knowledge on structured interventional strategy, physical activity, sedentary activity, and healthy dietary pattern regarding prevention of atherosclerotic risk factors among middle age adolescence in school setting.

Design: School-based pre-experimental one group pre-post test time series research design.

Setting/Participants: A total of 150 middle age adolescence between age of 15 – 17 years, who are studying in Higher Secondary Schools during the study period belonging to 10th to 12th standard at Arakonam, Vellore district, Tamil Nadu, India.

Study Period: Data will be collected in March – August 2021, and intervention duration is 3 months.

Intervention: Structured interventional strategy on knowledge regarding prevention of atherosclerotic risk factors by video with power point presentation, lesson with information booklet on sedentary activity, dietary habits, and physical activity by 3 phases; **1st phase (1 – 4 weeks):** skipping and walking including warm-up and resting for 30 minutes once in a week; **2nd phase (5 – 8 weeks):** skipping, walking, running, and dancing including warm-up and resting for 45 minutes twice a week; **3rd phase (9 – 12 weeks):** skipping, walking, running, dancing, and bicycling including warm-up and resting for 60 minutes thrice a week.

Main outcome measure: Primary outcomes will be assessed at 4th week, 8th week, and 12th week post intervention are BMI and physical activity. Secondary outcomes are dietary intake and sedentary activity will be assessed at 4th week, 8th week, and 12th week post intervention.

Conclusions: Structured interventional strategy can improve primary and secondary outcomes among middle age adolescence.

Discussion: This study will provide whether integration of knowledge on Atherosclerotic risk factors improved by structured interventional strategy, promoting good dietary habit messages to prevent atherosclerotic risk factors, and how to decrease secondary activity. Physical activity levels are remarkably very low among middle age adolescents, this study provides feasible with low cost intervention to be effective in schools, the learned behavior can be implemented throughout the life of an individuals and also sharing their experience with peers, family members and part of their community.

1. Introduction

Atherosclerosis is a systemic disease, and also known as Arteriosclerosis or hardening of the arteries, in which lipid, or fatty deposits, plaque, inflammation, cells, and scar tissue build up within the walls of small and large arteries supply to a variety of end-organs, it includes mainly the heart, brain, kidneys, and extremities (Benjamin et al., 2017). There are several risk factors, which is mainly focused on traditional and non-traditional (Martin-Timon et al., 2014) traditional risk factors are obesity (Nadeau et al., 2014), overweight, physical inactivity, active and passive smoking (Steinberger et al., 2016), tobacco use, consumption of alcohol, high caloric diets, sedentary lifestyle, stress, hypertension, diabetes mellitus, dyslipidemia, sleep apnea, lack of estrogen in women, urbanization, and modernization (Al Makadma, 2017; Martin-Timon et al., 2014; Smeltzer et al., 2010). Non-traditional risk factors are family history/genetics (Jeemon et al., 2017), aging, gender (more often in male than females), race/ethnicity, hyperinsulinemia, postprandial hyperglycemia, glucose variability, thrombogenic factors, inflammation C-reactive protein, and homocysteine (Martin-Timon et al., 2014; Smeltzer et al., 2010). One of the associated factor lower socio-economic status during adolescence significantly predicted greater adult CVD risk through the following pathways: health behaviors, financial stress, lack of medical/dental care, and educational attainment (Doom et al., 2017). Study reported that higher systolic blood pressure, and low-density cholesterol, and lower level of high-density cholesterol in young adulthood associated with atherosclerosis and high carotid intima-media thickness (Benjamin et al., 2017).

An adolescent may consider as an individual in the 10-19 years and categorized age group into three stages: early (~10 - 14years), middle (~15 - 17years), and late adolescence (~18 - 21years) (World Health Organization, 2020) Around 1.2 billion people of the world's population are adolescents aged 10 to 19 years (World Health Organization, 2017). Adolescence is a critical and complex, often considered healthy period of life, and time of remodeling of the brain's reward system (Patton et al., 2016). It is a period of transitional changes takes place in biological, physical, psychological, socio-cultural, and emotional changes, and considers very important foundation for the future of both individuals and nations (Taylor et al., 2016; World Health Organization, 2020). It is a time period where many characteristics of good, positive health are at their height, they may have fewer needs than those in early childhood or in later years (Patton et al., 2016; Taylor et al., 2016).

In middle to late adolescence stage, many cognitive abilities increase markedly, and start to decrease before the age of early 30s. In this phase in adolescent brain development brings various skills like self-regulatory, intellectual, decision making, and problem solving skills leading to greater future orientation (Patton et al., 2016). Adolescent health is influenced by their environment, education, supportive relationships, stakeholders, and access to high quality health services. We must create the opportunities to meaningfully engage with them in all aspects of their healthy lives, therefore reduce the adolescent health risks for later-life non-communicable diseases will require an unprecedented extent of coordination across sectors from the global to the local level (Patton et al., 2016).

Education is a powerful determinant of adolescent health to create knowledge, changing their attitudes towards positive spectrum of health, and decrease the burden of cardiovascular risks in adult life. Through education as an individual they can extend their life expectancy with less ill health (Patton et al., 2016). Educational strategy to reduce the burden of atherosclerosis, to be focused on multidisciplinary lifestyle intervention pneumonic based on "DREAM" which includes healthy diet, relaxation, physical exercise, increase activity and reduce screen time (Lewis et al., 2017), yoga and meditation to decrease stress, increase self-concept for adolescents and aiming to improve long-term health and wellbeing (Mayr et al., 2020). Previous study reported that there was an decreased in fast food consumption but non-significant regarding unhealthy snacks among adolescents and concluded that adequate higher dose of interventions may be needed in achieving the goal effectively (Mohammadifard et al., 2013). Systematic review stated that to reduce risk of CVD including atherosclerosis, strongly linked to behavioural factors, and self-efficacy to make healthy lifestyle choices in adolescents (Smith et al., 2015). Poor healthy diet, sedentary behavior, and lack of physical activity which rise to more number of complications such as obesity, overweight, metabolic syndrome, and hypertension, all those factors contributing to CVD in early adulthood (Nikolic et al., 2011).

The researcher wants to select a younger, healthier generation of middle age adolescence in school, which is a suitable place to initiate primordial prevention (Catalano et al., 2012). So the school will be an ideal setting, with unique individual's to promote positive healthy life choices, enhancing knowledge on preventive measures, transform values and information to the individuals, families, and community (Al Makadma, 2017).

Due to rapid technological advancement there is marked reduction in physical exercise, unhealthy food choices, and increased screen time among the adolescents needed different target behaviors that require different intervention strategies. The benefits of school-based interventions on moderate to vigorous physical activity, improve healthier dietary pattern, and decrease sedentary screen time among adolescents include captive audience and wide reach of target group (Hynynen et al., 2016). On the other hand the rights of children obligations states that to recognize the special health and development needs and rights of adolescents and young people (World Health Organization, 2017, 2020). CVDs typically occur in middle age or later, risk factors are determined to a great extent by behaviors learned in childhood and continued into adulthood. Adolescence provides an opportunity for teenagers to incorporate healthy lifestyle behaviors that will benefit them not only during the teenage years, but also throughout the life span.

1.1 Need and Significance of Study

Promoting healthy behaviour during adolescence, and taking steps to better protect young people from CVD risks are critical for the prevention of health problems in adulthood, and for countries' future health and ability to develop and thrive (World Health Organization, 2017). The Rashtriya Kishor Swasthya Karyakram is the National Adolescent Health Programme (NAHP) addresses on to promote behaviour change in adolescents to prevent non-communicable diseases such as hypertension, stroke, cardiovascular diseases and diabetes (Satia, 2018).

The role of community health nurse is expanding and extending care to achieve the Sustained Development Goals (SDGs) 2030 agenda "Leaving no one behind", target – 3 emphasizes to ensure healthy lives and promote well – being for all ages, a 25% relative reduction in risk of pre mature mortality from cardiovascular diseases, cancer, diabetes or chronic renal disease and 10% relative reduction in prevalence of insufficient physical activity (United Nations, 2019).

As a nurse, she can lobby for health promotive measures and mobilize community resources for conducting education and research programme for identifying risk factors among adolescents regarding atherosclerosis. As an educator/research investigator can envisage information, education, and communication to students, colleagues and media regarding risk factors and its preventive strategies. Researcher can empower the community to build up relationship between adolescent and stakeholders, work together, for the achievement of common goals. The overall concept is to develop an integrated programme for the prevention and control of non-communicable diseases as part of primary health care system. The experience of the researcher working in community observed that intervention studies to prevent and cut-off risk factors for atherosclerosis are inadequate; hence the researcher has selected the following study.

Keeping in view of the above-mentioned information, the personal experience of the researcher and the discernment that lecture cum discussion with power point presentation on atherosclerotic risk factors and its prevention, physical activity such as rope skipping, running, stretching exercises through physical education teacher and dietary modification through distribution of pamphlets and monthly once newsletter to parents for enhancing and motivating the adolescence in day to day activities, and will cause a significant reduction in the incidence of the disease, motivated the researcher to conduct structured interventional strategy among the middle age adolescence regarding prevention of atherosclerotic risk factors.

1.2 Statement of the Problem

Prevention of Atherosclerotic risk factors by Structured Interventional Strategy on knowledge, physical activity, and dietary pattern among middle age adolescence in selected Schools at Arakonam, Tamilnadu, India.

1.3 Objective of Study

1.3.1 Aim of study

The main aim of this study is to evaluate the effectiveness of structured interventional strategy on knowledge, physical activity, and dietary pattern among middle age adolescence in selected schools.

1.3.2 Principal Objectives

1. To assess the prevalence of atherosclerotic risk factors among middle age adolescence.

2. To assess the level of existing knowledge on prevention of atherosclerotic risk factors, physical activity, sedentary activity, and dietary pattern among middle age adolescence.

3. To evaluate the effectiveness of structured interventional strategy on knowledge regarding prevention of atherosclerotic risk factors among middle age adolescence.

4. To evaluate the effects of physical activity, sedentary activity, and healthy dietary pattern regarding prevention of atherosclerotic risk factors among middle age adolescence.

4. To compare the relationship between knowledge, physical activity, sedentary activity, and healthy dietary pattern regarding prevention of atherosclerotic risk factors among middle age adolescence.

5. To find the association between pre and post test knowledge, physical activity, sedentary activity, and healthy dietary pattern regarding prevention of atherosclerotic risk factors with selected demographic variables among middle age adolescence.

1.4 Research Questions

The study is aimed to answer the following questions:

1. What is the prevalence among middle age adolescence in selected schools in Arakonam, Tamilnadu, India?

2. Is there any previous knowledge, physical activity, sedentary activity, and dietary pattern regarding prevention of atherosclerotic risk factors?

3. How could be structured interventional strategy and physical activity improved; reduction in sedentary activity and changes in healthy dietary pattern on prevention of atherosclerotic risk factors among middle age adolescence.

1.5 Operational Definitions

Effectiveness: It refers to the outcome in the knowledge, physical activity, sedentary activity, and dietary pattern score after structured interventional strategy, daily physical activity, reduction screen time, and changes in healthier dietary pattern by structured and modified standardized questionnaire.

Structured interventional strategy: It refers to written and verbal materials used for teaching with assistance of video, which is prepared by the researcher. It is intended to provide information on knowledge, physical activity, sedentary activity, and dietary pattern regarding atherosclerosis and its prevention among middle age adolescence.

Knowledge: It refers to the written responses of the middle age adolescence regarding prevention of atherosclerotic risk factors measured by a structured questionnaire prepared by the researcher which includes general information, risk factors, symptoms, diagnosis, and prevention. The score '1' is given for right answer and '0' given for wrong answer. The total score is '40'. The composite score for knowledge will be calculated by adding the scores for all domains and rescaling it within100. Scores will then recoded into three categories: Inadequate knowledge: score considered ? 50%; moderately adequate knowledge: score considered 51 - 75%; adequate knowledge: score considered 76 - 100%.

Physical activity: It refers to physical exercise done by middle age adolescents measured by modified standardized physical activity questionnaire. It consists of 8-items value from 1 to 5 for each items (items 1 to 8) used in the physical activity composite score, simply take the mean of these 8 items, which results in the final PAQ - A activity summary score. A score of '1' indicates low physical activity, whereas a score of '5' indicates high physical activity (Kowalski et al., 2005).

Dietary pattern: It refers to food habits of middle age adolescents measured by modified standardized adolescent food habit checklist (AFHC). It consists of 20-items, 1 point for each 'healthy' response. Final score should be adjusted for 'not applicable' and missing responses using the formula: AFHC score = no of 'healthy' responses x (20/no of items completed) (Johnson et al., 2002).

Sedentary activity: It refers to any waking behavior characterized by an energy expenditure less than 1.5 METs while in a sitting or reclining posture e.g., watching television/video, working on a computer for or homework, active commuting to school, Sitting around (chatting with friends / on the phone/chilling) (Hardy et al., 2007; Lewis et al., 2017).

Atherosclerosis: Atherosclerotic is a disease condition in which plaque builds up inside the arteries (Smeltzer et al., 2010).

Atherosclerotic Risk factors: It refers to the factors responsible for the development of Atherosclerosis of blood vessels in the middle age adolescence. The risk factors are un healthy blood cholesterol levels, high blood pressure, smoking, insulin resistance, diabetes, overweight or obesity, lack of physical activity, older age, family history of early heart disease, high levels of C-reactive protein (CRP), high levels of triglycerides, sleep apnea, stress and alcohol. Prevalence of Atherosclerotic risk factors is measured by general appearance, anthropometric measurements, and bio-physiological measurement.

Middle age adolescence: It refers to boys and girls studying from 10th to 12th standards between the age group of 15 to17 years among selected schools at Arakonam, Tamilnadu.

Schools: It refers to higher secondary schools having students from 10th to 12th standard.

1.6 Outcome

1.6.1 Primary outcomes

It will be assessed at 4th week, 8th week, and 12th week post intervention are BMI and physical activity.

1.6.2 Secondary outcomes

Dietary intake and sedentary activity will be assessed at 4th week, 8th week, and 12th week post intervention.

1.7 Limitations

Ø The study is limited to the middle age adolescence group of 10th to 12th standard only.

Ø The study is limited to the 3 higher secondary schools of Aarokonam, Vellore district, Tamilnadu, India.

2. Literature Review

Study conducted in rural district of West Midnapore, India to assess the level of health awareness of CVD in adolescent school-aged children 14–16 years age, with the goal of establishing school-based health education and development of heart-healthy lifestyle practices. Result shown that the total participants 2995 (48% response rate) from 20 schools with the mean age 14.7 years, 46% were male, 53% were in the 9th grade, and the rest were in the 10th grade. Students' awareness assessed in six domains with a maximum score of 100, the mean pre-test score was 41.1 (SD -10.5) and the mean post-test score was 48.1 (SD - 16.9) ($p < 0.001$). Concluded that awareness among the adolescent children on CVD and its risk factors were far from optimal. A school-based educational program may help increase awareness and reduce the future burden on CVD in the community (Ray et al., 2016).

A cross-sectional prospective study corroborated in West Bengal, India were examined the prevalence of obesity, and hypertension in school children 8 to 18 years of age of either gender, attending 14 public schools and analyzed to identify potential risk factors. Result showed that the prevalence of pre-hypertension was 13.43% (95% CI ¼ 12.74% -14.12%), hypertension 4.05% (95% CI ¼ 6.43% - 7.47%), and abnormal body mass index was and 38.67 (95% CI ¼ 37.68%-39.66%). The prevalence rates children and adolescents 8 to 18 years of age, those 13 to 18 years had significantly more high risk factors such as hypertension, and obesity (Chaudhury et al., 2017).

A study assessed to determine the association between Mediterranean diet adherence, physical activity patterns (i.e., screen time and PA after school), and weight status among 605 Chilean school children for cardio-respiratory fitness test. Heart rate (HR), systolic (SBP) and diastolic blood pressures (DBP) were assessed before and immediately after the 20 minutes shuttle run test and the changes induced were calculated pre and post test. Results showed that the cardiovascular response was higher in obese than normal weight and overweight children in terms of HR ($P = 0.007$) and SBP ($P < 0.001$). The variation in pre / post test SBP presented inverse association with WC ($P = 0.026$), food habits ($P <0.001$), PA after school ($P < 0.001$) and PA patterns ($P < 0.001$) and HR reported inverse association with PA patterns ($P = 0.029$). The study concluded that school children with obesity showed a higher cardiovascular response in HR and SBP than normal weight and overweight peers (Delgado-Floody et al., 2020).

A study evaluated the association of parental smoking exposure (PSE) with the secretion of adipocyte-derived hormones and cardio metabolic risk factors in 3150 Chinese children aged 6-18 years. Six adipokines related to insulin resistance and metabolic syndrome (MetS) were measured. PSE was reported in nearly two-thirds of the children.PSE was independently associated with increases of 39.2% in leptin and 3.9% in retinol binding protein-4 and decreases of 11.4% in fibroblast growth factor 21 and 4.6% in adiponectin levels ($p < 0.05$ for all), risks for central obesity ($p<0.0001$), elevated blood pressure ($p = 0.028$) and MetS ($p = 0.006$). The associations of PSE with hypertension and MetS were abolished when adjusted for adiposity parameters or the above-mentioned adipokine profiles (Li et al., 2020).

A pre –post design evaluated the effects of a lifestyle intervention on markers of oxidized lipoproteins in 35 obese Latino youth, mean age15.5±1.0 years, BMI percentile 98.5±1.2 with pre diabetes of 12-week lifestyle intervention targeting improvements in nutrition and increases in physical activity. Intervention resulted in reductions in weight ($p=0.042$), BMI and BMI percentile ($p=0.001$), body fat ($p=0.025$), waist circumference ($p=0.025$), fasting insulin ($p=0.008$), triglycerides ($p=0.032$), total cholesterol ($p=0.002$), VLDL-cholesterol ($p=0.029$), HDL- cholesterol ($p=0.022$), non-HDL ($p=0.007$), and intake of fruits and vegetables ($p=0.025$), oxidized LDL significantly decreased after the intervention ($p=0.022$) while oxidized HDL significant increased ($p=0.056$). Concluded that the utility of lifestyle intervention to improve the atherogenic phenotype of Latino youth who are at high risk for developing premature cardiovascular disease and type 2 diabetes (Renteria-Mexia et al., 2019).

A study evaluated the relationships of parenting characteristics and academic achievement in adolescence in relation to ideal cardiovascular health in midlife men. Adolescent parenting measures revealed a single better parenting was significantly related to more ideal cardiovascular health in Blacks only ($p = .004$), academic achievement was related to ideal cardiovascular health, ? = '.13, but was no longer significant after controls for adult socio-economic status (SES). Adult SES was a strongly correlated of ideal cardiovascular health in both Black and White men. Black men exposed to positive parenting during adolescence had more ideal cardiovascular health based on American Heart Association guidelines. Improving academic achievement in adolescence may benefit adult cardiovascular health, indirectly through improving adult SES (Matthews et al., 2017).

Cigarette smoke is understood by in vitro, in vivo, and epidemiological studies to have acute and sub acute effects leading to cardiovascular consequences. The specific mechanistic domains include acute effects on endothelial function, platelet function, vasoconstriction, autonomic function, heart rhythm, and inflammation. Sub acute effects can include inflammation via oxidative stress, dyslipidemia, thrombosis, insulin sensitivity, and endothelial dysfunction. Nicotine alone is associated with hemodynamic alterations, dyslipidemia and insulin resistance. Acrolein causes oxidative stress, inflammation and is linked to hypertension, dyslipidemia, arrhythmia, and thrombosis while crotonaldehyde is an atherogenic compound that induces plaque instability, increases thrombosis, and may have direct negative inotropic effects. Cadmium is documented to cause inflammation and facilitate atherosclerosis. Lead exposure may cause hypertension and predicts cardiovascular mortality. Various particulate matter is known to be arrythmogenic and precipitate CVD events (Raghuveer et al., 2016).

Adequate arterial function includes the transmission of blood flow to downstream tissue capillary beds with minimal energy loss, and regulation of blood flow in those tissue beds with steady flow proportional to metabolic demand. These arterial actions are determined by the structure and function of large "conduit" and small "resistant" arteries. Assessment of structure includes, but is not limited, to measurement of carotid intima media thickness (CIMT), and arterial stiffness. SHS exposure appears to distort arterial structure. These distortions are of clinical relevance as recent reports indicate that peripheral artery disease is higher in those exposed to second hand smoke in childhood adjusted for other adult predictors of peripheral artery disease (Priest et al., 2014).

The prevalence of short sleep duration in 244 healthy high school students and the relevance of early risk factors to cardiovascular disease in adulthood suggest that adolescence is an opportune time to evaluate links between sleep duration and cardiovascular disease risk. We examined associations among actigraphy assessed sleep duration and sleep debt with elevated C - reactive protein (CRP), a known risk factor for cardiovascular disease. Participants in the High Risk CRP Group had significantly higher BMIs (88.09% ± 18.9) compared to participants in the Low to Moderate Risk CRP Group (77.16% ± 22.86; $p<.01$). Higher BMIs were associated with higher high sensitivity CRP levels ($p<.001$). There was a trend for higher parental education in the Low to Moderate Risk CRP Group (44.1% with some college or college degree) compared to 27.3% with education beyond high school in the High Risk CRP Group ($p<.07$). Short sleep duration in adolescence influences inflammation and its downstream consequences to cardiovascular risk will be critical to risk stratification and intervention. That these relationships may be observed prior to the onset of clinical or even subclinical disease suggests that adolescence may provide opportunities for disease prevention (Hall et al., 2015).

Data were obtained from 1477 families participating in the Child Health Check Point study, nested within the longitudinal study conducted among Australian Children Investigated whether neighborhood and family socioeconomic position (SEP), measured biennially from ages 0 to 1 year onward, was associated with carotid intima–media thickness (IMT) at ages 11 to 12 years.Disadvantaged family and neighborhood SEP was cross-sectionally associated with thicker maximum carotid IMT The difference in maximum carotid IMT between the highest and lowest family SEP quartile measured at ages 10 to 11 years was 10.7 lm ($p=0.004$), adjusted for age, sex, pubertal status, passive smoking exposure, body mass index, blood pressure, and arterial lumen diameter. In longitudinal analyses, family SEP measured as early as age 2 to 3 years was associated with maximum carotid IMT at ages 11 to 12 years ($p=0.02$). Concluded that a robust association between lower SEP in early childhood and carotid IMT in mid-childhood (Liu et al., 2017).

This study was to estimate CV risk in apparently healthy adolescent girls and aimed to test some new, emerging CV risk factors and their interaction with the traditional ones, such as obesity. To assess the impact of low bilirubin levels, as a routine biochemical parameter, as an additional risk factor for atherosclerotic disease in the adult phase. 45 obese adolescent girls and 45 age- and sex-matched normal weight controls, all nonsmokers, mean age 17.8±1.22 years were included. Anthropometric and biochemical parameters were measured. Cardiovascular Risk Score (CVRS) was calculated by adding the points for each risk factor (e.g. sex, HDL-c, non-HDL-Cholesterol (HDL-c), blood pressure and fasting glycemia). Results stated that a significant positive relationship between CVRS and ALT, high sensitivity CRP and TG/HDL-c, but an opposite relationship between CVRS and total bilirubin were found (P<0.001). Higher waist circumference (WC) and LDL-c, but lower HDL-c were independent predictors of lower bilirubin values (P<0.001). Concluded that the obese adolescent girls increased risk of cardiovascular disease late in life. In addition to the traditional risk factors, total bilirubin may have the potential to discriminate between low and higher risk for cardiovascular disturbances in healthy adolescent girls (Klisic et al., 2016).

A prospective cohort Shanghai Women's Health Study (SWHS) to investigate adolescent exercise in association with cancer, cardiovascular (CVD), and all-cause mortality among middle-aged and older women. 74,941 Chinese women aged 40–70 years recruited 1996–2000. In-person interviews at enrollment assessed adolescent and adult exercise history, medical and reproductive history and other lifestyle and socioeconomic (SES) factors. Mortality follow-up occurs via annual linkage to the Shanghai Vital Statistics Registry. Result stated that adjusting for birth year and other adolescent factors, adolescent exercise was associated with reduced risk of CVD). Results were attenuated after adjustment for adult SES and lifestyle factors. Participation in sports teams was inversely associated with cancer mortality (95% CI): 0.86(0.76–0.97)). Joint adolescent and adult exercise was associated with reduced risk of all-cause and CVD mortality (HRs (95% CIs): 0.80(0.72–0.89) and 0.83(0.69–1.00). Concluded that adolescent exercise participation, independent of adult exercise, was associated with reduced risk of CVD, and all-cause mortality (Nechuta et al., 2015).

In the Young Finns Study Improvement in HDL-cholesterol:LDL-cholesterol ratio and obesity from childhood to adulthood was associated with reduced CIMT progression. However, carotid intima-media thickness (CIMT) still progressed more than in participants who had normal baseline and follow-up HDL-cholesterol: LDL-cholesterol ratio and BMI. Frequent fruit intake and physical activity in childhood were also associated with lower adult CIMT. Similarly, the number of CVD risk factors in childhood and adulthood were associated with increased pulse wave velocity in adulthood, but reduction in the number of risk factors and improvement in BMI over time were associated with lower pulse wave velocity in adulthood. Pediatric lifestyle remains important, these data also suggest that vascular changes in childhood can at least be improved with appropriate improvement in CVD risk factors during young adulthood (Juonala et al., 2010).

A prospective study corroborated to estimate population-level trajectory groups of life course cardiovascular risk to explore their impact on mid-life atherosclerotic and metabolic outcomes among Bogalusa Heart participants n=1,269, between the mean ages of 9.6–48.3 years, each with at least 4 study visits from childhood in 1973 through adulthood in 2016. Outcomes included mid-life subclinical atherosclerotic measures [(carotid intima-media thickness (cIMT), pulse wave velocity (PWV)], metabolic indicators [(diabetes and body mass index (BMI)], and short physical performance battery (SPPB). Adult metabolic and vascular outcomes were significantly determined by life course cardiovascular risk trajectory groups (all p<0.01). Those in the High-Low group had lower risks of diabetes (20% vs. 28%, respectively; p=.12) and lower BMIs (32.4 kg/m2 vs. 34.6 kg/m2; p=0.06) than those who remained at high risk (High-High) throughout life. However, the High-Low group had better cIMT (0.89 mm vs. 1.05 mm; p<.0001) and PWV (7.8 m/s vs. 8.2 m/s; p=0.03) than the High-High group. For all outcomes, those in the Low-Low group fared best. Concluded that considerable movement between low and high relative cardiovascular risk strata over the life course. Children who improved their relative cardiovascular risk over the life course achieved better mid-life atherosclerotic health despite maintaining relatively poor metabolic health through adulthood (Pollock et al., 2019).

A study to aimed to study the association between serum ferritin levels and dyslipidemia in adolescents, because dyslipidemia is considered an important modifiable cardiovascular risk factor in the young 1,879 subjects (1,026 boys and 853 girls) from the 2009–2010 Korean National Health and Nutrition Examination Survey IV. Subjects were categorized into quartiles according to their lipid parameters, which were classified according to age and gender. Those in the highest quartile groups for total cholesterol, low-density lipoprotein cholesterol (LDL-C), and triglyceride concentrations were diagnosed as having dyslipidemia. Those in the lowest quartile for high-density lipoprotein cholesterol (HDL-C) values were diagnosed with abnormal levels. Results stated in boys, total cholesterol, LDL-C, and triglyceride concentrations were significantly correlated with serum ferritin levels. In both boys and girls, serum ferritin levels were negatively associated with HDL-C values, even after adjusting for all covariates. Concluded that serum ferritin levels were significantly associated with major dyslipidemia parameters, more prominently in boys than in girls, and this association represents a cardiometabolic risk factor (Kim et al., 2016).

Review suggested that there is strong evidence supports the concept that precursors of adult CVD begin in childhood, with obesity as an important correlate of overall CVD risk. The clearest evidence comes from autopsy studies showing that coronary atherosclerotic lesions occur in early life and are strongly associated with pediatric obesity, hypertension, and dyslipidemia. Obesity and CVD risk factors tend to persist over time. Observations from pediatric epidemiology studies over the past several decades further document that obesity, atherosclerosis, and associated risk factors begin in childhood. Lifestyle patterns, such as poor eating behavior, also begin early and influence CVD risk. The question of whether childhood obesity leads to increased adulthood CVD via increased CVD risk factors in childhood or via persistence into adulthood obesity, or both, remains unclear. However, sufficient data exist to warrant both obesity prevention and reduction in youth and adults (Nadeau et al., 2014).

The pathogenesis and progression of cardiovascular diseases are thought to be exacerbated by stress. Basic research indicates that the Transcendental Meditation technique produces acute and longitudinal reductions in sympathetic tone and stress reactivity. In adolescents at risk for hypertension, the technique has been found to reduce resting and ambulatory blood pressure, left ventricular mass, cardiovascular reactivity, and to improve school behavior. Research on adults with mild or moderate essential hypertension has reported decreased blood pressure and reduced use of anti-hypertensive medication. The technique has also been reported to decrease symptoms of angina pectoris and carotid atherosclerosis, to reduce cardiovascular risk factors, including alcohol and tobacco use, to markedly reduce medical care utilization for cardiovascular diseases, and to significantly decrease cardiovascular and all-cause morbidity and mortality. These findings have important implications for inclusion of the Transcendental Meditation program in efforts to prevent and treat cardiovascular diseases and their clinical consequences (Barnes & Orme-Johnson, 2012).

3. Research Methodology

3.1 Research approach: Quantitative Research Approach

3.2 Research design: Pre-experimental one group pre-post test time series research design.

O_1 x_1 O_2 x_2 O_3 x_3 O_4

O_1 - Observation at the pre-test (Baseline)

x_1 - Structured interventional strategy, Physical activity (skipping and walking including warm-up and rest) for 30 minutes, reduction in sedentary activity and healthy dietary habit tips by booklet and newsletter to parents.

O_2 - Observation after 4 weeks

x_2 - Structured interventional strategy, Physical activity (skipping, walking, running, and dancing including warm-up and rest) for 45 minutes, reduction in sedentary activity and healthy dietary habit tips by booklet and newsletter to parents.

O_3 - Observation after 8 weeks

x_3 - Structured interventional strategy, Physical activity (skipping, walking, running, dancing, and bicycling including warm-up and rest) for 60 minutes, reduction in sedentary activity and healthy dietary habit tips by booklet and newsletter to parents.

O_4 - Observation after 12 weeks

3.3 Interventional Components

Interventional components	Dose	Descripti
**Students**		
**Structured interventiona strategy**	Monthly once for 3 months x 60 minutes	Participants will attend point presentation o Atherosclerotic risk fact the researcher.
**Enhanced physical activity session**	**1st Phase (1 – 4 weeks):**<br>Skipping and walking including warm-up and resting for 30 minutes once in a week.<br>**2nd Phase (5 – 8 weeks):**<br>Skipping, walking, running, and dancing including warm-up and resting for 45 minutes twice a week<br>**3rd Phase (9 – 12 weeks):**<br>skipping, walking, running, dancing, and bicycling including warm-up and resting for 60 minutes thrice a week	Sport sessions will b physical education teac schools. Behavioral m reinforced during the co
**Sedentary activity**	Monthly once for 3 months x 30 minutes<br>Ø	Lesson with Information<br>Encourage for active

		school;<br><br>Ø Information about wh[illegible] change/reduce screen t[illegible]<br><br>Ø Be active with friends and[illegible]
**Dietary Pattern**	Monthly once for 3 months x 30 minutes	Lesson with Information[illegible]<br><br>Ø Nutrients to build a health[illegible]<br><br>Ø Healthy food choic[illegible] atherosclerosis risk fact[illegible]<br><br>Ø Increase fruits and vegeta[illegible]<br><br>Ø Cut, serve, taste and[illegible] vegetables with class m[illegible] members,<br><br>Ø Drink more water, [illegible] sweetened beverages, a[illegible]
**Parents**		
Newsletter	Monthly once for 3 months	Parents of study partici[illegible] three newsletters conta[illegible] on the potential co[illegible] excessive screen-use a[illegible]<br><br>Middle age adolescent[illegible] reducing screen-based [illegible] home, and tips for healt[illegible] also be provided with t[illegible] baseline information.

3.4 Study Variables

The following table explains the variables and measurement tools will be used in the current study.

Type of Variables	Variables to be measured	Instrument used to[illegible]
**Dependent Variables**	Ø Knowledge of adolescents regarding the prevention of atherosclerosis risk factors	Structured questionnaire<br><br>to assess knowledge

	Ø Physical activity	Adolescent Physical Activity c
	Ø Sedentary activity	Adolescent Sedentary Activity
	Ø Dietary habits	Adolescent Food Habit Check
**Independent Variables**	Ø Structured interventional strategy (Video with Power Point Presentation, Lesson with Information booklet on sedentary activity and dietary habits)<br>Ø Physical Exercise	Ø Structured teaching plan, developed by the researcher<br>Ø By physical education teacher
**Demographic Variables**	Age in years, gender, order of born, type of family education, religion, locality of residence, monthly income of parents (in rupees), occupation of father and mother educational status of father and mother, family history, and source of information on Atherosclerosis	Structured questionnaire investigator

3.5 Setting:

The study setting is selected schools at Arakonam, Vellore District, Tamil Nadu State, India.

3.6 Population

All adolescents who are 15 to 17 years of age, studying in 10th to 12th standard in Higher Secondary Schools are the population for the present study.

3.6.1 Target Population

It refers to all middle age adolescent students who are studying in Higher Secondary Schools during the study period belonging to 10th to 12th standard at Tamil Nadu.

3.6.2 Accessible Population

It refers to the aggregate of adolescents with whom the designated criteria are conformed and accessible to the investigator. Thus, the accessible population is 3 Higher Secondary Schools, a total number of adolescents who are studying in 10th to 12th standards at Arakonam, Vellore district, Tamil Nadu.

3.7 Sampling

3.7.1 Sample

In the current study, the adolescents who are studying in 10th to 12th standards who fulfilled the inclusive criteria are the study participants.

3.7.2 Sample selection criteria

Inclusion criteria

v Middle age adolescence who are studying in higher secondary schools aged between 15-17 years at Arakonam.

v Middle age adolescence who are willing to participate in the study

v Able to understand English and Tamil

v Both genders are included in the study

v BMI above 50th percentile

Exclusion criteria

v Middle age adolescence who are sick

v Middle age adolescence who are practicing in weight management

v Middle age adolescence whose sibling or relatives studying in other schools will be selected for the study

v Middle age adolescence who is below 50th percentile or underweight

3.7.3 Sampling technique

Probability stratified random sampling technique will be used for this study. In stratified random sampling, the strata are formed based on individuals' shared attributes or characteristics. Simple random sampling technique (lottery method) will be used to select 3 schools from Arakonam, Vellore District. From the above population 150 samples will be selected from 3 Higher Secondary Schools. 50 samples will be selected from each school. Each class as 5-6 divisions' consisting of 45-50 students in each section. One section from each class is randomly selected 15-20 samples who fulfilled the inclusion criteria will be taken as a sample from the particular class. Thus, 150 students are selected from class 10, 11 and 12th standards.

3.7.4 Sample size

Sample size was calculated by Power analysis based on the Pilot study.

3.8 Development of tool

The investigator used the following steps for preparation of the tools for the study

• Extensive literature review

• Preparation of the blue print for the tools

• Consultation with experts from the field of study

• Preparation of the final draft of the tools

• Editing of the tools

3.9 Description of the tools

Part I: Questionnaire to assess demographic characteristics of the middle age adolescents. It includes age in years, gender, order of born, type of family, education, religion, locality of residence, monthly income of parents (in rupees), occupation of father and mother, educational status of father and mother, family history, and source of information on Atherosclerosis.

Part II: Checklist to assess the prevalence of Atherosclerosis in middle age adolescents

Part III: A structured questionnaire to assess knowledge on prevention of Atherosclerotic risk factors among middle age adolescents

Part IV: Adolescent physical activity questionnaire (APAQ) to assess physical activity among middle age adolescence for last 7 days

Part V: Adolescent Food Habit Checklist (AFHC) to assess the food habits among middle age adolescence

Part VI: Adolescent Sedentary Activity Questionnaire (ASAQ) to assess the sedentary activity among middle age adolescence for weekdays and weekends

3.10 Score Interpretation

Part I

The information on demographic data to be collected from the middle age adolescence on 15 variables and this was not scored but it is used for descriptive analysis.

Part II

These tools included; general appearance of the subjects, anthropometric measurement, and bio-physiological measurements. For Anthropometric measurements, the cut off value for height, weight, BMI and waist circumference will be calculated as per standard recommended by Indian Academy of Pediatrics (Revised IAP Growth Charts for Height, Weight, BMI and Waist circumference for 5 to 18 years old Indian Children). The blood pressure will be assessed on centile values of Indian standard (IAP).

1. Scoring for Height in cm and Weight in Kg for boys and girls

< 25 Percentile - Below normal

50th Percentile - Normal

75th Percentile - Normal

90th Percentile - Below abnormal (action point to prevent obesity)

97th Percentile - Abnormal

2. Scoring cut off value for BMI for boys and girls

The following formula use to calculate Body Mass Index (BMI)

BMI = Weight (kg) / Height (m^2)

< 25th percentile - Underweight

50th Percentile - Normal

75th percentile - Normal

85th Percentile - Overweight (action point to prevent obesity)

95th percentile - Obese

3. Scoring cut off value for Waist Circumference for boys and girls

< 25th percentile - Underweight

50th Percentile - Normal

75th percentile - Overweight (action point to prevent obesity)

95th percentile - Obese

Scoring for Bio - Physical measurements

4. Scoring cut off value for systolic pressure and diastolic pressure for boys and girls

< 25th percentile - Hypotension

50th Percentile - Normal

75th percentile - Pre hypertension (action point to prevent hypertension)

95th percentile - Hypertension

Part III

It consists of 40 items. Each question had three answers with only one correct answer. When the response is correct 'one' mark was given and for the wrong answer 'zero'. The knowledge covered areas like general information of atherosclerosis, risk factors, symptoms, diagnosis and prevention of risk factors.

Scoring for Knowledge

SI.No.	Areas of Knowledge	No. of Items	Sc[illegible]
1	General Information	9	[illegible]
2	Risk factors	10	1[illegible]
3	Symptoms	8	[illegible]
4	Diagnosis	3	1[illegible]
5	Prevention of risk factors	10	[illegible]
	**Total**	**40**	4[illegible]

SCORING

Total item = 40, Total score 40 = 100%

<20 items: <50% Inadequate Knowledge

21 – 30 items: 51 – 75% Moderate Knowledge

>31 items: - >75% Adequate Knowledge

Part IV: Physical Activity Questionnaire among Middle Age Adolescence (Kowalski et al., 2004)

SCORING

1) Item 1 (Spare time activity)

- Take the mean of all activities ("no" activity being a 1, "7 times or more" being a 5) on the activity checklist to form a composite score for item 1.

2) Item 2 to 7 (PE, lunch, right after school, evening, weekends, describes you best)

- The answers for each item start from the lowest activity response and progress to the highest activity response

- Simply use the reported value that is checked off for each item (the lowest activity response being a 1 and the highest activity response being a 5).

3) Item 8

- Take the mean of all days of the week ("none" being a 1, "very often" being a 5) to form a composite score for item 8.

4) Item 9

- Can be used to identify students who had unusual activity during the previous week, but this question is **NOT** used as part of the summary activity score. As a researcher will refer the subjects to the health centers with the stakeholders.

5) How to calculate the final PAQ-A activity summary score

- Once you have a value from 1 to 5 for each of the 8 items (items 1 to 8) used in the physical activity composite score, you simply take the mean of these 8 items, which results in the final PAQ - A activity summary score.

A score of 1 indicates low physical activity, whereas a score of 5 indicates high physical activity.

Part V: Food Habit Questionnaire among Middle Age Adolescence (Johnson et al., 2002)

SCORING

1 point for each 'healthy' response. Final score should be adjusted for 'not applicable' and missing responses using the formula: AFHC score = no of 'healthy' responses x (20/no of items completed).

Part VI: Sedentary Activity Questionnaire among Middle Age Adolescence (Hardy et al., 2007)

SCORING

Time spent in each category of sedentary behavior.

Total time being sedentary will be calculated for weekdays, weekend days and all days.

The values then characterized as poor, fair, and good practice.

3.11 Conclusion

A high prevalence of cardiovascular disease risk factors and their disproportional distribution among the study population indicated an inevitable risk of cardiovascular events in near future.

Increased awareness and improved primary care services may decrease the incidence of coronary artery disease and improve overall quality of life (Ahmed et al., 2017). Overweight, excess body fat, lipid profile, sedentary behavior, and history of CVD in family were the most prevalent cardiovascular risk factors among adolescents. The adolescents had higher rates of overweight and excess fat. As for the stages, a higher percentage of individuals with sedentary behavior, overweight, total cholesterol and LDL in comparison with other stages. Individuals with changes in nutritional status were more likely to develop hypertension, changes in total cholesterol, LDL, triglycerides, insulin, and low HDL when compared to healthy individuals. Concluded that the cardiovascular risk factors have been observed in younger and younger individuals and are important factors to identify a population at

risk (do Prado et al., 2015). Cardiovascular risk factors are highly prevalent among school children. Importantly, school children lack adequate knowledge regarding cardiovascular risk factors. School based interventions are required for cardiovascular risk reduction in childhood.

3.12 References

Ahmed, A. M., Hersi, A., Mashhoud, W., Arafah, M. R., Abreu, P. C., Al Rowaily, M. A., & Al-Mallah, M. H. (2017). Cardiovascular risk factors burden in Saudi Arabia: The Africa Middle East Cardiovascular Epidemiological (ACE) study Cardiovascular risk factors burden in Saudi Arabia. *Journal of the Saudi Heart Association*, *29*(4), 235–243. https://doi.org/10.1016/j.jsha.2017.03.004

Al Makadma, A. S. (2017). Adolescent health and health care in the Arab Gulf countries: today's needs and tomorrow's challenges. *International Journal of Pediatrics and Adolescent Medicine*, *4*, 1–8. https://doi.org/10.1016/j.ijpam.2016.12.006

Barnes, V. A., & Orme-Johnson, D. W. (2012). Prevention and treatment of Cardiovascular disease in adolescents and adults through the Transcendental Meditation® program: a research review update. *Curr Hypertens Rev*, *8*(3), 227–242. https://www.ncbi.nlm.nih.gov/pmc/articles/PMC3624763/pdf/nihms412728.pdf

Benjamin, E. J., Blaha, M. J., Chiuve, S. E., Cushman, M., Das, S. R., Deo, R., Sarah D. de Ferranti, Floyd, J., Fornage, M., Gillespie, C., Isasi, C. R., Jiménez, M. C., Lori Chaffin Jordan, Judd, S. E., Daniel Lackland, Lichtman, J. H., Lisabeth, L., Simin Liu, Chris T. Longenecker, … Muntner, P. (2017). Heart disease and Stroke statistics —2017 update: a report from the American Heart Association. *Circulation*, *135*(10), e146–e603. https://doi.org/CIR.0000000000000485.

Catalano, R. F., Fagan, A. A., Gavin, L. E., Greenberg, M. T., Irwin, C. E., Ross, D. A., & Shek, D. T. L. (2012). Worldwide application of prevention science in adolescent health. *The Lancet*, *379*(9826), 1653–1664. https://doi.org/10.1016/s0140-6736(12)60238-4

Chaudhury, A. R., Reddy, T. V., Divyaveer, S. S., Patil, K., Bennikal, M., Karmakar, K., Chatterjee, S., Dasgupta, S., Sircar, D., & Pandey, R. (2017). A Cross-sectional prospective study of asymptomatic urinary abnormalities, blood pressure, and body mass index in healthy school children. *Kidney International Reports*, *2*, 1169–1175. https://doi.org/10.1016/j.ekir.2017.07.018

Delgado-Floody, P., Alvarez, C., Caamaño-Navarrete, F., Jerez-Mayorga, D., & Latorre-Román, P. (2020). Influence of Mediterranean diet adherence, physical activity patterns, and weight status on cardiovascular response to cardiorespiratory fitness test in Chilean school children. *Nutrition*, *71*, 1–6. https://doi.org/10.1016/j.nut.2019.110621

do Prado, P. P., de Faria, F. R., de Faria, E. R., Franceschini, S. do C. C., & Priore, S. E. (2015). Cardiovascular risk and associated factors in adolescents. *Nutricion Hospitalaria*, *32*(2), 897–904. https://doi.org/10.3305/nh.2015.32.2.8824

Doom, J. R., Mason, S. M., Suglia, S. F., & Clark, C. J. (2017). Pathways between childhood/adolescent adversity, adolescent socioeconomic status, and long-term cardiovascular disease risk in young adulthood. *Social Science and Medicine*, *188*, 166–175. https://doi.org/10.1016/j.socscimed.2017.06.044

Hall, M. H., Lee, L., & Matthews, K. A. (2015). Sleep duration during the school week is associated with C-reactive protein risk groups in healthy adolescents. *Sleep Medicine*, *16*(1), 73–78. https://doi.org/10.1016/j.sleep.2014.10.005.Sleep

Hardy, L. L., Booth, M. L., & Okely, A. D. (2007). The reliability of the Adolescent Sedentary Activity Questionnaire (ASAQ). *Preventive Medicine*, *45*(1), 71–74. https://doi.org/10.1016/j.ypmed.2007.03.014

Hynynen, S. T., Van Stralen, M. M., Sniehotta, F. F., Araújo-Soares, V., Hardeman, W., Chinapaw, M. J. M., Vasankari, T., & Hankonen, N. (2016). A systematic review of school-based interventions targeting physical activity and sedentary behaviour among older adolescents. *International Review of Sport and Exercise Psychology*, *9*(1), 22–44. https://doi.org/10.1080/1750984X.2015.1081706

Jeemon, P., Harikrishnan, S., Sanjay, G., Sivasubramonian, S., Lekha, T. R., Padmanabhan, S., Tandon, N., & Prabhakaran, D. (2017). A PROgramme of Lifestyle Intervention in Families for Cardiovascular risk reduction (PROLIFIC study): design and rationale of a family based randomized controlled trial in individuals with family history of premature coronary heart disease. *BMC Public Health*, *17*, 1–11. https://doi.org/10.1186/s12889-016-3928-6

Johnson, F., Waddle, J., & Griffith, J. (2002). The adolescent food habits checklist: reliability and validity of a measure of healthy eating behaviour in adolescents. *European Journal of Clinical Nutrition*, *56*(7), 644–649. https://doi.org/10.1038/sj.ejcn.1601371

Juonala, M., Viikari, J. S. A., Kähönen, M., Taittonen, L., Laitinen, T., Hutri-Kähönen, N., Lehtimäki, T., Jula, A., Pietikäinen, M., Jokinen, E., Telama, R., Räsänen, L., Mikkilä, V., Helenius, H., Kivimäki, M., & Raitakari, O. T. (2010). Life-time risk factors and progression of carotid atherosclerosis in young adults: the cardiovascular risk in young finns study. *European Heart Journal*, *31*(14), 1745–1751. https://doi.org/10.1093/eurheartj/ehq141

Kim, Y. E., Kim, D. H., Roh, Y. K., Ju, S. Y., Yoon, Y. J., Nam, G. E., Nam, H. Y., Choi, J. S., Lee, J. E., Sang, J. E., Han, K., & Park, Y. G. (2016). Relationship between serum ferritin levels and dyslipidemia in Korean adolescents. *PLoS ONE*, *11*(4), 1–12. https://doi.org/10.1371/journal.pone.0153167

Klisic, A., Kavaric, N., Soldatovic, I., Bjelakovic, B., & Kotur-Stevuljevic, J. (2016). Relationship between cardiovascular risk score and traditional and nontraditional cardiometabolic parameters in obese adolescent girls. *Journal of Medical Biochemistry*, *35*(3), 282–292. https://doi.org/10.1515/jomb-2016-0005

Kowalski, K. C., Crocker, P. R. E., Columbia, B., & Donen, R. M. (2004). *The Physical Activity Questionnaire for Older Children (PAQ-C) and Adolescents (PAQ-A) Manual*. *August*.

Kowalski, K. C., Crocker, P. R. E., & Rachel M. Donen. (2005). The Physical Activity Questionnaire for older Children (PAQ-C) and Adolescents (PAQ-A) manual. In *College of Kinesiology, University of Saskatchewan*. papers://305a9bed-f721-4261-8df7-5414758c1624/Paper/p910

Lewis, B. A., Napolitano, M. A., Buman, M. P., Williams, D. M., & Nigg, C. R. (2017). Future directions in physical activity intervention research: expanding our focus to sedentary behaviors, technology, and dissemination. *Journal of Behavioral Medicine*, *40*(1), 112–126. https://doi.org/10.1007/s10865-016-9797-8

Li, Y., Wang, D., Wang, Y., Zhao, Y., Han, L., Zhong, L., Zhang, Q., Speakman, J. R., Li, M., & Gao, S. (2020). Impact of parental smoking on adipokine profiles and cardiometabolic risk factors in Chinese children. *Atherosclerosis*, *301*(March), 23–29. https://doi.org/10.1016/j.atherosclerosis.2020.03.023

Liu, R. S., Mensah, F. K., Carlin, J., Edwards, B., Ranganathan, S., Cheung, M., Dwyer, T., Saffery, R., Magnussen, C. G., Juonala, M., Wake, M., Burgner, D. P., Olds, T., Baur, L., Gold, L., Lycett, K., Kerr, J. A., & Davies, S. (2017). Socioeconomic position is associated with carotid intima-media thickness in mid-childhood: the longitudinal study of Australian children. *Journal of the American Heart Association*, *6*(8), 1–16. https://doi.org/10.1161/JAHA.117.005925

Martin-Timon, I., Sevillano-Collantes, C., Segura-Galindo, A., & Canizo-Gomez, F. J. del. (2014). Type 2 diabetes and cardiovascular disease: have all risk factors the same strength? *World Journal of Diabetes*, *5*(4), 444–470. https://doi.org/10.4239/wjd.v5.i4.444

Matthews, K. A., Boylan, J. M., Jakubowski, K. P., Cundiff, J. M., Lee, L., Pardini, D. A., & Jennings, J. R. (2017). Socioeconomic status and parenting during adolescence in relation to ideal cardiovascular health in black and white men. *Health Psychology*, *36*(7), 673–681. https://doi.org/10.1037/hea0000491

Mayr, H. L., Cohen, F., Isenring, E., Soenen, S., & Marshall, S. (2020). Multidisciplinary lifestyle intervention in children and adolescents - Results of the project GRIT (Growth, Resilience, Insights, Thrive) pilot study. *BMC Pediatrics*, *20*, 1–16. https://doi.org/10.1186/s12887-020-02069-x

Mohammadifard, N., Sarrafzadegan, N., Ghassemi, G. R., Nouri, F., & Pashmi, R. (2013). Alteration in unhealthy nutrition behaviors in adolescents through community intervention: Isfahan Healthy Heart Program. *ARYA Atherosclerosis*, *9*(1), 89–97.

Nadeau, K. J., Maahs, D. M., Daniels, S. R., & Eckel, R. H. (2014). Childhood obesity and cardiovascular disease: links and prevention strategies. *Nature Rev Cardiology*, *8*(9), 513–525. https://doi.org/10.1038/nrcardio.2011.86.

Nechuta, S. J., Shu, X. O., Yang, G., Cai1, H., Gao, Y.-T., Li, H.-L., Xiang, Y.-B., & Zheng, W. (2015). Adolescent exercise in association with mortality from all causes, cardiovascular disease, and cancer among middle-aged and older Chinese women. *Cancer Epidemiol Biomarkers Prev*, *24*(8), 1270–1276. https://doi.org/10.1158/1055-9965.EPI-15-0253

Nikolic, I. A., Stanciole, A. E., & July, M. Z. (2011). *Health, Nutrition and Population (HNP) discussion paper chronic emergency: why NCDs matter*. https://openknowledge.worldbank.org/bitstream/handle/10986/13591/639270WP0Chron0Box0361533B00PUBLIC0.pdf?sequence=1&isAllowed=y/Accessed 10 Sep 2020

Patton, G. C., Sawyer, S. M., Santelli, J. S., Ross, D. A., Afifi, R., Allen, N. B., Arora, M., Azzopardi, P., Baldwin, W., Bonell, C., Kakuma, R., Kennedy, E., Mahon, J., McGovern, T., Mokdad, A. H., Patel, V., Petroni, S., Reavley, N., Taiwo, K., … Viner, R. M. (2016). Our future: a Lancet commission on adolescent health and wellbeing. *The Lancet*, *387*(10036), 2423–2478. https://doi.org/10.1016/S0140-6736(16)00579-1

Pollock, B. D., Stuchlik, P., Harville, E. W., Mills, K. T., Tang, W., Chen, W., & Bazzano, L. A. (2019). Life course trajectories of cardiovascular risk: Impact on atherosclerotic and metabolic indicators. *Atherosclerosis*, *280*, 21–27. https://doi.org/10.1016/j.atherosclerosis.2018.11.008

Priest, J. R., Nead, K. T., Wehner, M. R., Cooke, J. P., & Leeper, N. J. (2014). Self-reported history of childhood smoking is associated with an increased risk for peripheral arterial disease independent of lifetime smoking burden. *PLoS ONE*, *9*(2), e88972. https://doi.org/10.1371/journal.pone.0088972

Raghuveer, G., White, D. A., Hayman, L. L., Hayman, L. L., Woo, J. G., Villafane, J., Celermajer, D., Ward, K. D., Ferranti, S. D. de, & Justin Zachariah. (2016). Cardiovascular consequences of childhood secondhand tobacco smoke exposure?: prevailing. *Circulation*, *134*(16), e336–e359. https://doi.org/10.1161/CIR.0000000000000443.Cardiovascular

Ray, M., Guha, S., Ray, M., Kundu, A., Ray, B., Kundu, K., Goswami, S., Bhatt, D. L., Selker, H. P., & Goldberg, R. J. (2016). Cardiovascular health awareness and the effect of an educational intervention on school-aged children in a rural district of India. *Indian Heart Journal*, *68*, 43–47. https://doi.org/10.1016/j.ihj.2015.10.302

Renteria-Mexia, A., Vega-Lopez, S., Olson, M. L., Swan, P. D., Lee, C. D., Williams, A. N., & Shaibi, G. Q. (2019). Effects of a lifestyle intervention on markers of cardiometabolic risk and oxidized lipoproteins among obese adolescents with prediabetes. *Public Health Nutrition*, *22*(4), 706–713. https://doi.org/10.1017/S1368980018003476

Satia, J. (2018). Challenges for adolescent health programs: what is needed? *Indian Journal of Community Medicine*, *43*(5), S1–S5. https://doi.org/10.4103/ijcm.IJCM_331_18

Smeltzer, S. C., Brenda G. Bare, Janice L. Hinkle, & Kerry H. Cheever. (2010). Textbook of Medical-Surgical Nursing. In *Lippincott Williams & Wilkins* (12th ed.).

Smith, C. F., Heneghan, C., & Ward, A. (2015). Moving focus from weight to health. What are the components used in interventions to improve cardiovascular health in children? *PLoS ONE*, *10*(8), 1–13. https://doi.org/10.1371/journal.pone.0135115

Steinberger, J., Daniels, S. R., Hagberg, N., Isasi, C. R., Kelly, A. S., Lloyd-Jones, D., Pate, R. R., Pratt, C., Shay, C. M., Towbin, J. A., Urbina, E., Van Horn, L. V., & Zachariah, J. P. (2016). Cardiovascular health promotion in children: challenges and opportunities for 2020 and beyond: a scientific statement from the American Heart Association. *Circulation*, *134*(12), e236–e255. https://doi.org/10.1161/CIR.0000000000000441

Taylor, S. A., Borzutzky, C., Jasik, C. B., Mihalopoulos, N. L., Smith-Barron, K., Woolford, S. J., Garber, A., McPherson, M., AlBuhairan, F. S., Kohn, M., Garland, B. H., & Dixon, A. (2016). Preventing and treating adolescent obesity: a position paper of the society for adolescent health and medicine. *Journal of Adolescent Health*, *59*(5), 602–606. https://doi.org/10.1016/j.jadohealth.2016.08.020.

United Nations. (2019). *Transforming our world: the 2030 agenda for sustainable development*. sustainabledevelopment.un.org/ Accessed 23 August 2019.

World Health Organization. (2017). Global Accelerated Action for the Health of Adolescents (AA-HA!) guidance to support country implementation. In *Who*. https://apps.who.int/iris/bitstream/handle/10665/255415/9789241512343-eng.pdf?sequence=1%0Ahttp://www.searo.who.int/indonesia/topics/global_accelerated_action_for_the_health_of_adolescents_(aa-ha!).pdf%0Ahttp://apps.who.int/iris/bitstream/10665/255415/1/9

World Health Organization. (2020). *Orientation programme on adolescent health for health- care providers, Department of Child and Adolescent health and Development*. http://www.who.int/child-adolescent-health/ Accessed 28 July 2020.
