# Supplementary material for: Effectiveness of structured interventional strategy for middle-aged adolescence (SISMA-PA) for preventing atherosclerotic risk factors—A study protocol
Source: PLoS One. 2022 Jul 19;17(7):e0271599. doi: 10.1371/journal.pone.0271599 (PMC9295980; doi:10.1371/journal.pone.0271599)
Supplement: Attachment — Submitted filename: Response to reviewers.docx [file pone.0271599.s006.docx]

**Date: May 2022**

# POINT-BY-POINT RESPONSE LETTER

PLOS ONE

# Manuscript ID PONE-D-22-02489 Title: Effectiveness of structured interventional strategy for middle-aged adolescence (SISMA-PA) for preventing atherosclerotic risk factors: a study protocol

# We express our sincere appreciation to the reviewers and editor for their time, comments, suggestions, and interest in this study protocol. The reviewers and editor had pointed out constructive comments, and legitimate questions which improved the paper.

Please find our responses below:

***Note: Correction and edition made in the track change version:***

|  | **(Reviewer-1) Review questions/comments** | **Response** |
| --- | --- | --- |
| **1** | The major weakness of this protocol is that the author/s highlighted cost-effectiveness in the background and it should be the main objective of the proposed study, but the author/s did not say anything of how they will calculate cost. Authors must address this issue in the data analyses. | **Yes, Thank you for your comments and time consideration.**  **We have mentioned the rationale for cost-effectiveness under the discussion section now. Page 12, Line 241-243.**  **Rationale:**  **This intervention will require no special training by paid coaches, and will be enhanced by selecting low-cost seasonal fruits and vegetables.** |
|  | **(Reviewer-2) Review questions/comments** | **Response** |
| **1.** | Why the investigators think that 12-week is enough to improve the awareness on prevention of atherosclerotic risk factors by structured interventional strategy, physical activity training, information on a healthy diet, and reducing sedentary activity? Usually, lifestyle-related diseases need longer intervention because of the relactance of participates against lifestyle change. | **Thank you, for your comments and time consideration.**  **We have mentioned the rationale under the methods section, interventions. Page 07, Line 134-135.**  **Rationale:**  **We strongly believe that 12 weeks of intervention will yield a 50-75% dosage response based on previous studies [34–36] of minimum clinically important difference.** |

With regards

Gomathi Munusamy

[gomathilingeswaran2678@gmail.com](mailto:gomathilingeswaran2678@gmail.com)
